# Beyond COVID-19, the case for collecting, analysing and using sex-disaggregated data and gendered data to inform outbreak response: a scoping review

**DOI:** 10.1136/bmjgh-2024-015900

**Published:** 2025-01-15

**Authors:** McKinzie Gales, Emelie Love Yonally Phillips, Leah Zilversmit Pao, Christine Dubray, Clara Rodriguez Ribas Elizalde, Shirin Heidari, Marie-Amelie Degail, Marie Meudec, M Ruby Siddiqui, Simone E Carter

**Affiliations:** 1 Global Health Center, Centers for Disease Control and Prevention, Atlanta, Georgia, USA; 2 Epidemiology Intervention and Training Department, Epicentre, Paris, France; 3 Centers for Disease Control and Prevention, Atlanta, Georgia, USA; 4 World Health Organization, Geneva, GE, Switzerland; 5 Graduate Institute of International and Development Studies, Geneve, GE, Switzerland; 6 Health Emergencies Programme, World Health Organization, Geneve, GE, Switzerland; 7 Public Health, Institute of Tropical Medicine, Antwerpen, Antwerpen, Belgium; 8 Integrated Outbreak Analytics, UNICEF, London, UK; 9 PUBLIC HEALTH EMERGENCIES, UNICEF, Kinshasa, Congo (the Democratic Republic of the)

**Keywords:** Global Health, Maternal health, Public Health, Infections, diseases, disorders, injuries, Systematic review

## Abstract

**Introduction:**

Understanding sex and gender differences during outbreaks is critical to delivering an effective response. Although recommendations and minimum requirements exist, the incorporation of sex-disaggregated data and gender analysis into outbreak analytics and response for informed decision-making remains infrequent. A scoping review was conducted to provide an overview of the extent of sex-disaggregated data and gender analysis in outbreak response within low- and middle-income countries (LMICs).

**Methods:**

Five databases were searched for peer-reviewed literature examining sex- and gender-specific outcomes for communicable disease outbreaks published in English between 1 January 2012 and 12 April 2022. An adapted version of the WHO’s Gender Analysis Matrix was used to synthesise evidence, which was then mapped across four phases of the outbreak timeline: prevention, detection, treatment/management and recovery.

**Results:**

71 articles met inclusion criteria and were included in this review. Sex-, gender-, and pregnancy-related disparities were identified throughout all four phases of the outbreak timeline. These disparities encompassed a wide range of risk factors for disease, vulnerability, access to and use of services, health-seeking behaviour, healthcare options, as well as experiences in healthcare settings and health and social outcomes and consequences.

**Conclusion:**

Significant gender-evidence gaps remain in outbreak response. Evidence that is available illustrates that sex and gender disparities in outbreaks vary by disease, setting and population, and these differences play significant roles in shaping outbreak dynamics. As such, failing to collect, analyse or use sex-disaggregated data and gendered data during outbreaks results in less effective responses, differential adverse health outcomes, increased vulnerability among certain groups and insufficient evidence for effective prevention and response efforts. Systematic sex- and gender-based analyses to ensure gender-responsive outbreak prevention, detection, treatment/management and recovery are urgently needed.

**WHAT IS ALREADY KNOWN ON THIS TOPIC**

**WHAT THIS STUDY ADDS**

- This scoping review synthesises the existing evidence on how sex and gender intersect with disease outbreaks and response activities across all aspects of the outbreak response timeline in various contexts, highlighting key gaps in the availability and utilisation of sex-disaggregated data and gender analyses.

**HOW THIS STUDY MIGHT AFFECT RESEARCH, PRACTICE OR POLICY**

- This study emphasises the necessity of integrating sex and gender aspects in outbreak analytics, planning and response as a matter of routine. It identifies sex- and gender-specific aspects, including pregnancy-related implications, for each phase of the response, providing valuable guidance for future research, informing practice and shaping policy decisions in the field of outbreak response. Additionally, this study highlights the need to address barriers to collecting, analysing and using sex-disaggregated data and gendered data to improve response efforts that are measured by the reduction of morbidity and mortality.

## Introduction

 Understanding the impact of sex and gender on outbreak dynamics is critical to delivering an effective and equitable outbreak response. Integrating sex and gender aspects in outbreak analytics and response is essential for identifying potential gendered patterns of transmission, affected populations and devising appropriate control strategies.^1^,^5^ Nonetheless, during disease outbreaks, sex and gender aspects continue to be overlooked leading to catastrophic and enduring repercussions.^2 6 7^

While the terms ‘sex’ and ‘gender’ are often used interchangeably, there are important distinctions between the terms. Sex is a classification system, defined by various biological traits such as chromosomes, reproductive organs and hormone profiles. These differences impact biological susceptibilities and physiological responses to pathogens, shaping exposure and transmission patterns.^8^ Gender encompasses sociocultural constructed norms, roles, behaviours and relations that are considered appropriate for women and girls, men and boys within a society.^9^,^11^ Gender-related factors can influence exposure to pathogens, access to information, resources and services, and self-efficacy and empowerment. Gender, intersecting with factors such as socioeconomic status (SES), race, ethnicity, sexual orientation and age, contribute to health disparities and health and social outcomes during outbreaks.^6^ Recognising the distinction between sex and gender and systematically collecting data on both allow for a comprehensive analysis of health issues, including how biological differences intersect with social and cultural factors to shape health experiences and outcomes.

International standards and guidelines increasingly emphasise sex- and gender-inclusive approaches to health, as can be seen in the UN Sustainable Development Goals^12^ and the WHO’s 13th general programme of work,^13^ both of which recognise the need to apply a gender lens to achieving improved health outcomes. Several resources and tools are emerging to support researchers and response actors to improve the integration of sex and gender into health research and programming. The Sex and Gender Equity in Research (SAGER) guidelines, published in 2016, provide a systematic approach to considerations of sex and gender differences in research across disciplines.^10^

Endorsed by numerous academic journals and, recently recognised by WHO, the SAGER guidelines are fostering improved transparency in sex and gender reporting in research, to bridge the gender evidence gap.^14^ Additionally, toolkits such as the World Bank’s Gender in Preparedness and Response Toolkit^15^ and WHO’s Incorporating Intersectional Gender Analysis into Research on Infectious Diseases of Poverty Toolkit^16^ have been developed to support the integration of gender into specific aspects of public health emergency preparedness and response. Despite these international standards and guidelines, significant gaps persist in the availability and use of sex-disaggregated and gendered data during outbreaks and public health emergencies, and appeals have been made to adapt the SAGER guidelines to address these gaps.^7 17 18^

### Aims and objectives

This systematic literature review aims to synthesise and assess the scope of the peer-reviewed evidence on sex, gender and/or pregnancy during outbreaks in low- and middle-income countries (LMICs) published between 2012 and 2022, explore the implications of integrating sex-disaggregated data and gendered data in the outbreak and public health emergency analytics and responses in LMICs, and describe current gaps in data collection, analysis and use of these data in outbreak analytics and responses. By addressing these objectives, this review aims to underscore the importance of collecting, analysing and using sex-disaggregated data and gendered data in the outbreak response efforts and highlight the areas that require further research.

## Methods

We conducted a scoping literature review following the Preferred Reporting Items for Systematic Reviews and Meta-Analyses scoping review guidelines.^19^ The review process consisted of the steps described in online supplemental figure 1.

### Search strategy

A search strategy was employed on five electronic databases (Medline/PubMed, Embase, Global Health, Scopus and Global Index Medicus), using the following search terms: ‘sex’ or ‘gender’ alongside ‘disease outbreak*’, ‘emergency response’, ‘disaster response’, ‘global health’, ‘humanitarian crisis’ or ‘humanitarian emergency*’ (see online supplemental table A). This search was conducted within titles, abstracts and keywords of studies published in English from 1 January 2012 and 12 April 2022. Additionally, a backward citation search was conducted to identify further relevant studies.

Covidence^20^ was used to manage the search results, including the removal of duplicate articles. Predetermined criteria were used to review titles and abstracts. Inclusion criteria were as follows: peer-reviewed articles; present original data or secondary analysis; focus on human subjects; contain relevant keywords for sex, gender and pregnancy; be based in an LMIC as per the 2022 World Bank List^21^; report on an outbreak related to diseases listed by the Sphere Standards^22^; and/or by the International Health Regulation’s declaration of public health emergencies of international concern.^23^ Pregnancy was searched separately from sex or gender as it has both biological and sociocultural relevance in outbreak dynamics. The exclusion criteria included high- and middle-income country settings; research not considering sex, gender or pregnancy; articles that did not specify outbreak, epidemic or pandemic for diseases endemic to the study setting; and literature published in languages other than English. Additionally, due to extensive existing research and confounding factors such as changes in the level and mechanisms of response during declared pandemics and research focused on co-morbidities and secondary infections, COVID-19, HIV and tuberculosis were excluded from this study. Full texts of the remaining articles were independently screened by two investigators for eligibility, with conflicts resolved through consensus.

### Synthesis

A data extraction template was created and included the following variables: author, title, study location, disease, study design, objectives, study population, participant count including pregnant and non-pregnant women, and findings aligned with study aims including (1) evidence of differences in outbreak dynamics by sex, gender and pregnancy status; (2) implications for outbreak analytics and response across the outbreak response timeline including prevention, detection, treatment/management and recovery; and 3) gaps in data collection, analysis and use by sex, gender and pregnancy status listed in the literature. The data extraction template was piloted prior to implementation. Data from each of the 71 articles that met the specified inclusion criteria were extracted independently by two investigators and summarised through consensus.

### Analysis

The team used a modified version of the WHO’s Gender Analysis Matrix (table 1),^24^ applied across four phases of the outbreak response timeline: prevention, detection, treatment/management and recovery, to categorise sex, gender and pregnancy-status-related factors according to six health parameters: (1) risk and vulnerability to disease, (2) access to and use of services, (3) health behaviours and health-seeking patterns, (4) prevention, detection, treatment/management and recovery options, (5) experiences within the health system, and (6) health and social consequences. Evidence was grouped by data category (sex, gender, pregnancy) and overarching theme (ie, transmission, accessibility, stigma, etc) and disease, with specific evidence identified, country setting and reference listed. Evidence of differences in epidemiological measures of disease from sex-disaggregated data analyses was categorised under ‘sex’ unless linked to gender dimensions in the research. However, it should be noted that many of these observed differences in sex are likely driven by gender inequities. Evidence of differences between women and men, girls and boys linked in the article to sociocultural constructed norms, roles, behaviours and power relations were assessed using the WHO gender analysis framework and categorised under ‘gender’.^24^ Evidence of differences related to biological, physiological and sociocultural aspects of pregnancy were categorised under ‘pregnancy’.

**Table 1:** Modified version of the WHO’s gender analysis matrix

Response phase: (prevention, detection, treatment/management, recovery)					
**Datacategory**	**Themes**	**Disease**	**Evidence**	**Country**	**Firstauthor,year**
**Riskandvulnerability**					
Sex*					
**Gender†**					
**Pregnancy‡**					
**Access toanduse ofservices**					
**Sex**					
**Gender**					
**Pregnancy**					
**Healthbehavioursandhealth-seekingpatterns**					
**Sex**					
**Gender**					
**Pregnancy**					
**Prevention,detection,treatment/management,recoveryoptions**					
**Sex**					
**Gender**					
**Pregnancy**					
**Experiences within thehealth system**					
**Sex**					
**Gender**					
**Pregnancy**					
**Healthandsocialconsequences**					
**Sex**					
**Gender**					
**Pregnancy**					

* Identified disparities driven by sex-related biological/anatomical differences

† Identified disparities driven by gender-related norms, behaviours, roles and relations

‡ Identified disparities driven by physiological and sociocultural changes during pregnancy

### Patient and public involvement

While this scoping review was inspired by research on patient and public experiences and informed by actors working in outbreak response, patients and the public were not involved in the design, recruitment or conduct of this review.

## Results

### Characteristics of study

Out of 15 713 unique articles, 71 were included in this review. Study types included 23 reviews,^25^,^47^ 23 observational,^48^,^70^ 10 qualitative,^71^,^80^ 8 descriptive,^81^,^88^ 3 case-control,^89^,^91^ 2 incidence,^92 93^ 1 content analysis^94^ and 1 mixed-method.^95^

The disease outbreaks analysed included 31 Zika,^2629 33 34 36 37 42 46 50^,^53 58 63 71 74^ 17 ebola,^25 27 28 38 40 44 47 56 60 62 66 72 73 81 82 86 88^ 10 dengue,^42 48 54 55 57 64 68 87 92 93^ 6 hepatitis E,^30 31 61 67 70 89^ 5 influenza,^32 35 41 59 84^ 3 cholera,^39 49 90^ 3 yellow fever^43 65 91^ and 1 malaria.^45^ Five articles discussed more than one disease outbreak. Geographic distribution included 38 studies located in South America,^2629 33^,^37 39 40 42 43 46 47 50^ followed by 19 in West Africa,^2527 28 30 37^,^40 43^ 11 Central Africa,^3037^,^39 43^ 11 East Africa,^2730 38^,^40 44 45 60 86 89 90^ 11 South Asia,^31 37 39 44 45 48 59 61 68 70 84^ 9 North Africa,^2730^,^32 38 39 44 45 67^ 5 Central America,^37 46 53 72 74^ 4 South Africa,^30 31 35 39^ 4 Southeast Asia,^32 37 39 64^ 3 Caribbean,^37 46 51^ 1 Europe,^35^ 1 Middle East,^44^ 1 Oceania^45^ and 1 South Pacific.^36^ Seventeen articles covered more than one geographic region.

No studies reported data for people of diverse sexual orientation, gender diversity and expression.

### Prevention

The majority of the identified evidence on sex, gender and pregnancy-mediated outbreak dynamics related to the prevention phase of outbreak response. The complete adapted matrix showing evidence for sex, gender and pregnancy differences identified in the literature for each of the six health parameters applied to the prevention response phase, summarised below, can be found in online supplemental table B. Selected examples are displayed in table 2.

**Table 2:** Evidence of sex, gender and pregnancy status-related implications for prevention, selection of examples

Response phase: prevention					
Data category	Themes	Disease	Evidence	Country	First author, year
**RiskandVulnerability**					
**Sex**	Sexual transmission	Ebola	RNA in the semen for months post-recovery (prolonged potential for sexual transmission)	Guinea; Liberia; Sierra Leone; Other	Bebell *etal*^25^
**Gender**	Healthcare worker safety	Ebola	Midwives delivered infected babies without adequate PPE due to symptom absence	Sierra Leone	Bower *et al*^82^
**Pregnancy**	Epidemiological measures of disease	Influenza	Increased risk of severe disease in pregnant women due to altered respiratory and immune systems and increasingly attenuated inflammatory responses; greatest risk in the second and third trimesters	Arab Republic of Egypt; Indonesia; Malaysia; Vietnam; Other	Liu *et a*l^32^
**Access toanduse ofpreventionservices**					
**Sex**	*No evidence identified in the literature*				
**Gender**	Accessibility	Zika	Transportation barriers to access free female and male condoms	Ecuador	Casapulla *et al*^53^
**Pregnancy**	Availability	Zika	Restrictive policies related to SRH and abortions	Brazil	Coutinho *et al*^94^
**Preventionandprevention-seekingbehaviours**					
**Sex**	*No evidence identified in the literature*				
**Gender**	Risk perception	Ebola	Women reported engaging in a greater number of self-protective behaviours, perhaps as a result of greater perceived risk of exposure	Democratic Republic of Congo	Pham *et al*^66^
**Pregnancy**	Sociocultural norms	Zika	Condom use or abstinence during pregnancy is linked to infidelity/trust	Dominican Republic	Gurman *et al*^74^
**Preventionoptions**					
**Sex**	*No evidence identified in the literature*				
**Gender**	Gender-targeted response efforts	Ebola	Lack of prevention options targeted at safe hunting practices (greater impact on men)	Democratic Republic of Congo; Gabon; Guinea; Liberia; Republic of Congo; Sierra Leone; Sudan; Uganda	Nikangu *et al*^38^
**Pregnancy**	Unacceptable prevention options	Zika	Response recommendations to abstain from/postpone pregnancy ignored the fact that in many of the highest risk regions over half of pregnancies were unintended (due to gender inequalities resulting in a lack of autonomy over sexual and reproductive matters and limited access to contraception)	Brazil; El Salvador; Other	Johnson *et al*^29^
**Experiences within thehealth system**					
**Sex**	*No evidence identified in the literature*				
**Gender**	Trust	Zika	Women did not trust medical community’s capability to tackle virus due to the lack of agreement among medical providers	Brazil; other	Linde arias *et al*^71^
**Pregnancy**	Exclusion	Zika	Men feel excluded from reproductive decision-making in hospitals and excluded from attending prenatal visits	Dominican Republic	Gurman *et al*^74^
**Healthandsocialconsequences**					
**Sex**	*No evidence identified in the literature*				
**Gender**	Indirect consequences	Ebola	Greater secondary effects of response measures on women; increased domestic care responsibilities if schools are closed; increasing rates of gender-based violence	Brazil; Democratic Republic of Congo	Wenham *etal*^80^
**Pregnancy**	Maternal morbidity and mortality	Zika	Misalignment of response plans with sexual and reproductive policies resulted in spikes in clandestine and unsafe abortions with associated maternal mortality	Brazil	Borges *et al*^50^

#### Prevention risk and vulnerability

Prevention risk and vulnerability sex-disaggregated data showed differences in epidemiological measures of disease (ie, incidence, prevalence, severity and case fatality), sexual transmission and adverse events post-vaccination. For instance, ebola and Zika were found to persist in semen for months, which increased the risk of sexual transmission, with the highest risk found in male-to-female sexual transmission.^1825^,^27 37 42 44 46 53 71 79 92^ Gender disparities were identified in terms of pathogen exposure risk, autonomy in sexual and reproductive health (SRH) decisions and knowledge about infection prevention. Women frequently faced heightened disease exposure through caregiving and household activities,^2838^,^40 47 56 60 66 82 90 92^ while men exhibited greater exposure to infection outside the home.^31 38 39 48 60^ Furthermore, women, who dominate both paid and unpaid healthcare roles, faced increased ebola exposure risks due to inadequate access to suitable personal protective equipment (PPE) compared with men.^28 40 47 56 60 73 82^ Women’s lack of individual agency was linked to increased risk of sexually transmitted diseases due to intimate partner violence, challenges with condom negotiation^44 46 94^ and insufficient knowledge of disease prevention.^50 53 66 79^ Gender disparities were found to be compounded by SES, race and age for Zika risk.^74^,^7694^ Pregnancy has been found to introduce immunological and physiological changes, as well as changes in sociocultural norms, which can increase the risk of exposure, infection and adverse health outcomes among pregnant women. Compared with non-pregnant people, pregnant women have been found to experience higher rates of disease susceptibility, severe illness, complications and mortality, and face risks of adverse pregnancy, fetal and neonatal outcomes, as well as vertical transmission.^725^,^27 29^ SES, race/ethnicity and age were found to intersect with pregnancy status and shape sociocultural and structural barriers to reproductive health services relevant to disease prevention.^2530^,^32 35 45 54 57 61 62 64 67^

#### Access to and use of prevention services

The availability, accessibility and acceptability of prevention services varied across genders. For instance, the limited availability of contraception,^46^ geographical barriers and religious beliefs^53^ posed challenges for condom use in Zika prevention, disproportionately impacting women. Women also faced difficulties in accessing PPE during ebola outbreaks^55^ and clean water, sanitation and hygiene facilities during cholera and Zika outbreaks.^39 94^ In the context of Zika, affordability of prevention services and individual SES shaped access to repellents, mosquito nets, contraception and safe abortions, leaving the poorest women most at-risk of unintended pregnancies and Zika infection.^7 36 40 63 76 79 80^

#### Prevention behaviours and health-seeking patterns

Gender-driven disparities in decision-making, disease prevention tasks and perceived infection risks affected prevention behaviours. Studies examining women’s preventative behaviours during ebola and Zika found influences from family, friends, income and heightened self-perceived exposure risk.^53 66 74^ Gender norms around household responsibilities led to women shouldering most of the disease prevention and vector control responsibilities during Zika and ebola outbreaks.^71 74 75 94^ Zika prevention messaging often neglected men, resulting in minimal engagement in prevention.^94^ Reproductive intentions, risk perception and sociocultural norms shaped prevention behaviours among those who could become pregnant. For instance, Zika outbreaks impacted pregnancy intentions,^50 76^ while cultural and religious norms limited discussions on topics like sex and condom use during pregnancy in high-risk areas.^46^ Zika outbreaks resulted in an increased demand for legal and clandestine abortions linked to fears of microcephaly.^29 46 76 80 83^

#### Prevention options

Sex differences were evident in response strategies for preventing sexually transmitted Zika in women and men. The responsibility to abstain from sex or use barrier methods was placed on women.^37^ During ebola outbreaks, gender-sensitive response efforts neglected to educate men on safe hunting practices or women on safe caregiving practices.^38^ During many outbreaks, pregnant women faced limitations in prevention options. Historically, pregnant women were deemed ineligible to receive vaccines such as ebola, hepatitis E, and yellow fever.^27 31 65^ Although pregnant women are now eligible for these vaccines, contradictory messaging and insufficient safety evidence have hindered their uptake.^25 41 43 91^ Access to safe abortion services, a preventive measure against microcephaly due to Zika infection, was constrained by restrictive policies in many countries.^29 36 50 77 80^ Moreover, recommendations to postpone pregnancy during Zika outbreaks were impractical for individuals lacking SRH decision autonomy.^29 33 47^

#### Experiences with prevention services

Gender and pregnancy status shape healthcare experiences. Research on Zika found that women had limited prevention decision-making power^80^ and distrusted the medical community,^71^ while men were excluded from reproductive decision-making and prenatal visits.^74^ Pregnant women faced healthcare disparities in information, advice, exclusion, stigma and decision-making authority. They received inadequate information,^94^ with providers omitting Zika and microcephaly prevention options.^76 83^ Disparities were compounded by age and SES. For example, lower SES status women reported heightened stigma,^76^ affecting abortion choices,^46^ while higher SES women received more prevention information.^76^ Vaccine eligibility changes during ebola eroded pregnant women’s trust in preventive medicine.^66^

#### Health and social outcomes and consequences related to prevention

Prevention efforts had indirect consequences impacting women, resulting in disproportionate burdens during quarantine, heightened domestic care responsibilities and increased risk of gender-based violence.^47^ Discrepancies between prevention strategies and sexual and reproductive policies in outbreak-prone regions led to delayed pregnancies, rises in unsafe abortions, maternal deaths and fluctuations in birth rates during Zika.^36 37 50 72 83^ Furthermore, lower SES compounds sexual transmission risks and pregnancy and gender related Zika outcomes, amplifying risks of morbidity and mortality within lower SES groups.^76^

### Detection

The reviewed literature contained evidence on sex, gender and pregnancy-mediated outbreak dynamics related to the detection phase of outbreak response. The complete adapted matrix showing evidence for sex, gender and pregnancy differences identified in the literature for each of the six health parameters applied to the detection response phase, summarised below, can be found in online supplemental table C. Selected examples are displayed in table 3.

**Table 3:** Evidence of sex, gender and pregnancy status-related implications for detection, selection of examples from dengue

Response **phase**: **d**etection					
**Datacategory**	**Themes**	**Disease**	**Evidence**	**Country**	**Firstauthor,year**
**Detectionriskandvulnerability**					
**Sex**	*No evidence identified in the literature*				
**Gender**	*No evidence identified in the literature*				
**Pregnancy**	Pregnancy masking symptoms	Dengue	Clinical manifestations may resemble pregnancy-related conditions (hemoconcentration and haemolysis elevated liver enzymes low platelet count (HELLP) syndrome), complicating detection for physicians	Brazil	do Nascimento Einloft *et al*^54^
**Access toanduse ofscreening/diagnosticservices**					
**Sex**	*No evidence identified in the literature*				
**Gender**	*No evidence identified in the literature*				
**Pregnancy**	Availability	Zika	Limited testing and screening capacity in resource-poor settings may hinder detection of infection/exposed pregnant women, fetuses and infants	Brazil	Ambrogi *et al*^95^
**Diagnostic-seekingbehaviours**					
**Sex**	*No evidence identified in the literature*				
**Gender**	Detection bias	Dengue	Decreased detection and reporting among Asian women due to care-seeking from traditional practitioners	Pakistan	Aamir *et al*^48^
**Pregnancy**	Detection bias	Dengue	Higher incidence among pregnant women may be due to an increased use of formal healthcare services and prioritised screening	Brazil	Nascimento *et al*^87^
**Screening/diagnosticoptions**					
**Sex**	RNA detection	Zika	RNA detected in vaginal secretions after clearance from blood and urine	Colombia; Cuba; Dominican Republic; El Salvador; Guyana; Haiti; Honduras; Mexico; Nicaragua; Other	Vlassoff *et al*^46^
**Gender**	*No evidence identified in the literature*				
**Pregnancy**	Absence of diagnostic testing	Dengue	Routine neonatal diagnostic testing (PCR) is not conducted	Indonesia	Mulyana *et al*^64^
**Experiences withinscreening/diagnosticservices**					
**Sex**	*No evidence identified in the literature*				
**Gender**	*No evidence identified in the literature*				
**Pregnancy**	Delayed and/or misdiagnosis	Zika	Delayed or no diagnosis	Brazil; Dominican Republic	Brady *et al*^51^
**Healthandsocialconsequences ofdetection**					
**Sex**	*No evidence identified in the literature*				
**Gender**	*No evidence identified in the literature*				
**Pregnancy**	Blood transfusion risk	Zika	No licensed blood donor screening tests	Brazil; other	Marrs *et al*^33^

#### Detection risk and vulnerability

Pregnant women faced greater risk of underdiagnosis or misdiagnosis for ebola, dengue, and Zika. Case definitions can be more challenging to apply to pregnant women where broad definitions were linked to an overestimation of cases for ebola and Zika cases,^37 73^ while narrow case definitions were linked to underestimation.^52 88^ For example, fever-based definitions were estimated to miss over 70% of Zika cases in pregnant women.^52^ Additionally, clinical manifestations of a disease can resemble pregnancy-related symptoms, making detection challenging.^25 54 64 68^ In the West Africa Ebola outbreak, less than 1.5% of pregnant women with haemorrhagic or febrile symptoms were confirmed to have infection.^25^

#### Access and use of screening/diagnostic services

Limitations in available diagnostic tools have contributed to challenges in detecting diseases in pregnant women. In resource-poor settings, the lack of testing and screening capacity hindered identifying Zika infection in pregnant women, fetuses and infants.^37 51 95^

#### Diagnostic-seeking behaviours

Gender-related health-seeking behaviours were identified as contributing to detection bias in outbreaks. In some communities, differences in dengue detection between men and women revealed greater reliance of women on traditional practitioners operating outside of public surveillance systems.^48^ Conversely, higher detection of dengue and Zika among non-pregnant women aged 15 to 49^92^ and pregnant women were linked to higher frequency of SRH visits observed among these groups compared with men.^58 74 87 92^ However, in resource-limited settings, delayed presentation for healthcare likely resulted in an underestimation of pregnant women infected with ebola.^25^

#### Screening/diagnostic options

For some diseases, detectable viral RNA persisted longer in sex-specific and pregnancy-related fluids and tissues.^25^,^2737 44 53 71 72 75 81 88 92^ Zika RNA was detectable in semen even after clearance from blood and urine,^33 46^ and ebola RNA was detected in semen for up to 284 days^25 27 44 72^ and vaginal fluids/secretions up to 33 days post symptom onset.^25 27 44^ Pregnancy-related fluids had detectable ebola RNA levels despite negative blood test results.^27 81 88^ However, gaps persist in screening and diagnosis. Routine fetal and neonatal testing for dengue^64 68^ and ebola^25 62 81^ is not currently conducted. Testing for Zika and congenital Zika infection can be suboptimal^37 51^ and may cross-react with other viruses such as dengue, chikungunya and yellow fever.^33 46^ Microcephaly diagnosis presents challenges and may not be possible until the second trimester of pregnancy or up to 12 months after birth.^33 37 51 52 58 80 85^

#### Experiences with screening/diagnostic services

Pregnancy status affected diagnostic experiences, such as when pregnant women facing Zika encountered prolonged wait times for exams,^95^ misdiagnosis^79^ and delayed or no diagnosis.^51^ They also reported a lack of support, guidance and information regarding Zika testing and diagnosis from healthcare staff.^79^

#### Health and social outcomes of detection

Inadequate diagnostic and screening services contributed to disparities in health and social outcomes, particularly affecting pregnant women. In the context of Zika, high rates of asymptomatic cases posed challenges for detection in all populations. However, negative health and social outcomes of undiagnosed Zika were much more severe for pregnant women. Limited access to early pregnancy ultrasounds resulted in missed detection of Zika-related effects on fetal development, such as microcephaly.^37 95^ Furthermore, the lack of sufficient screening measures to prevent Zika transmission through blood transfusions disproportionately affected pregnant women, who are more likely to require blood transfusions.^33 79^

### Treatment/management

The reviewed literature contained evidence on sex, gender and pregnancy-mediated outbreak dynamics related to disease treatment/management. The complete adapted matrix showing evidence for sex, gender and pregnancy differences identified in the literature for each of the six health parameters applied to the treatment/management response phase, summarised below, can be found in online supplemental table D. Selected examples are displayed in table 4.

**Table 4:** Evidence of sex, gender and pregnancy status-related implications for treatment/management and selection of examples

Response **phase**: **t**reatment/**m**anagement					
**Datacategory**	**Themes**	**Disease**	**Evidence**	**Country**	**Firstauthor,year**
**Treatment/managementriskandvulnerability**					
**Sex**	*No evidence identified in the literature*				
**Gender**	*No evidence identified in the literature*				
**Pregnancy**	*No evidence identified in the literature*				
**Access toanduse oftreatment/managementservices**					
**Sex**	*No evidence identified in the literature*				
**Gender**	*No evidence identified in the literature*				
**Pregnancy**	Availability	Ebola	Fewer life-saving interventions available for pregnant women in Ebola treatment centres	Sierra Leone	Lyman *et al*^62^
**Treatment/management-seekingbehaviours**					
**Sex**	*No evidence identified in the literature*				
**Gender**	Time to treatment	Ebola	Time from initial symptoms to hospitalisation was shorter among women	Guinea; Liberia; Sierra Leone; South Sudan; Uganda	Gomes *et al*^27^
**Pregnancy**	Symptoms	Dengue	Roughly 76% of pregnant women sought treatment during the critical phase of infection	Indonesia	Mulyana *et al*^64^
**Treatment/managementoptions**					
**Sex**	*No evidence identified in the literature*				
**Gender**	*No evidence identified in the literature*				
**Pregnancy**	Clinical guidance	Dengue	No consensus on management of infection during pregnancy	Indonesia	Mulyana *et al*^64^
**Experiences in thehealth system**					
**Sex**	*No evidence identified in the literature*				
**Gender**	Gendered power dynamics	Ebola	Midwives had diverse experiences in outbreak care due to ICM restrictions; a midwife was fired for risking infection trying to improve care, but was later consulted by policymakers	Sierra Leone	Erland *et al*^73^
**Pregnancy**	Delayed treatment	Ebola	Pregnant women were denied maternal/obstetric care until a negative blood test which resulted in vesicovaginal fistula, intrauterine fetal death and maternal death	Sierra Leone	Erland *et al*^73^
**Healthandsocialconsequences oftreatment/management**					
**Sex**	*No evidence identified in the literature*				
**Gender**	Stigma	Ebola	Community’s lack of knowledge about ebola led to fear and stigma, affecting midwives (a midwife was evicted after the landlord found out she worked in an ebola treatment centre)	Sierra Leone	Erland *et al*^73^
**Pregnancy**	Morbidity and mortality	Ebola	Increased morbidity and mortality among women due to avoidance of health facilities	Nigeria	Fawole et al^56^

#### Treatment/management risk and vulnerability

No evidence was identified in the literature pertaining to sex, gender or pregnancy status and risk or vulnerability in treatment/management.

#### Access and use of treatment/management services

Pregnant women faced barriers to disease treatment during ebola and Zika outbreaks due to movement restrictions and disruptions in the availability, accessibility and acceptability of disease treatment and management during outbreaks.^40 46 72^ Limited interventions were available for pregnant women in ebola treatment centres.^62^

#### Treatment/management-seeking behaviours

Gender had a significant impact on treatment and management-seeking behaviours. Knowledge and awareness about treatment and management options, intervals between the onset of symptoms and seeking of formal care, and the type of care sought are influenced by gender. For influenza, women generally had higher treatment/management knowledge but relied more frequently on herbal medicine.^59^ In conflict-affected areas, women’s ability to seek healthcare during an ebola outbreak is further hindered by the risk of gender-based violence.^66^ For pregnant women, mistaking dengue- and ebola-related symptoms for pregnancy symptoms and fear of nosocomial transmissions limited treatment-seeking behaviours during outbreaks.^64 82^

#### Treatment/management options

Pregnant women, despite being a high-priority population for treatment, faced challenges to treatment and management options due to a lack of data on treatment safety and lack of consensus and clinical guidance for managing infectious diseases.^27 40 64 73 86^ Additionally, strict control measures in clinics impacted obstetric care, delaying and limiting access to timely specialised care and life-saving interventions for pregnant women admitted to ebola treatment centres.^25 62 73 88^ In some cases, pregnancy termination was accepted as the best option for maternal recovery from ebola.^70 73^ However, the availability of abortion services depended on national policies, healthcare provider compliance and acceptability to the patient.^76 83^

#### Experiences in healthcare

Gender-based discrimination and provider attitude contributed to differential treatment experiences between men and women. In dengue outbreaks, women faced delays in receiving hospital care and were admitted at later disease stages.^48^ Similarly, women reported inadequate care during Zika outbreaks.^95^ Pregnant women also encountered challenges in accessing proper treatment. Pregnant women infected with Zika reported experiencing stigma and prejudice from healthcare providers.^79^ Furthermore, pregnant women were denied essential maternal/obstetric care until they tested negative for ebola, which has been linked to adverse outcomes including vesicovaginal fistula, intrauterine fetal death and maternal mortality.^40 73^

#### Health and social outcomes of treatment/management

Gender impacts on treatment and management further shape health and social outcomes. During ebola outbreaks, midwives faced numerous serious health and social outcomes, including infection and death, being fired for making care decisions deemed to place staff at infection risk, experiencing community stigma and facing eviction during ebola.^73^ Additionally, women exhibited higher rates of morbidity and mortality as they avoided healthcare facilities during ebola outbreaks.^56^ Pregnant women faced severe disruptions in treatment and access to healthcare during ebola and Zika outbreaks, leading to decreased access to pre- and post-natal care, increased likelihood of unassisted deliveries and higher maternal mortality rates.^40 72^

### Recovery

The reviewed literature contained evidence on sex, gender and pregnancy-mediated outbreak dynamics related to the recovery phase of outbreak response. The complete adapted matrix showing evidence for sex, gender and pregnancy differences identified in the literature for each of the six health parameters applied to the recovery response phase, summarised below, can be found in online supplemental table E. Selected examples are displayed in table 5.

**Table 5:** Evidence of sex, gender and pregnancy status-related implications for recovery, selection of examples

Response **phase**: **r**ecovery					
**Datacategory**	**Themes**	**Disease**	**Evidence**	**Country**	**Firstauthor,year**
**Recoveryriskandvulnerability**					
**Sex**	Post-recovery sexual transmission	Zika	RNA has been detected in semen up to 370 days after onset of illness, but shedding of infective viral particles is rare after 30 days from the onset	Angola; American Samoa; Brazil; Columbia; Cuba; Dominican Republic; Ecuador; Guatemala; Guinea-Bissau; Haiti; Honduras; India; Jamaica; Nicaragua; Panama; Peru; Puerto Rico; Suriname; Thailand; Venezuela; Vietnam	Musso *et al*^37^
**Gender**	*No evidence identified in the literature*				
**Pregnancy**	Post-recovery transmission	Ebola	Increased risk for horizontal transmission during parturition post-maternal recovery	Guinea	Baggi *et al*^81^
**Access toanduse ofrecoveryservices**					
**Sex**	*No evidence identified in the literature*				
**Gender**	*No evidence identified in the literature*				
**Pregnancy**	Accessibility	Zika	Mothers often bear the burden of caring for children with CZS but face logistical barriers to obtaining medical services and welfare benefits for families with children with CZS (eg, transportation, documentation)	Brazil	Ambrogi *et al*^95^
**Health-seekingbehavioursduringrecovery**					
**Sex**	*No evidence identified in the literature*				
**Gender**	*No evidence identified in the literature*				
**Pregnancy**	Maternal health-seeking behaviours	Ebola	Reduced maternal health-seeking behaviours due to stigma and fear of nosocomial transmission	Nigeria	Fawole *et al*^56^
**Recoveryoptions**					
**Sex**	*No evidence identified in the literature*				
**Gender**	*No evidence identified in the literature*				
**Pregnancy**	Inadequate recovery services	Zika	Lack of medical knowledge, medications and specialised care for care of infants/children with CZS/microcephaly	Colombia	Tirado *et al*^79^
**Experiences withrecoveryservices**					
**Sex**	*No evidence identified in the literature*				
**Gender**	*No evidence identified in the literature*				
**Pregnancy**	*No evidence identified in the literature*				
**Healthandsocialoutcomes**					
**Sex**	*No evidence identified in the literature*				
**Gender**	Stigmatisation	Ebola	Stigmatisation of male survivors due to fears of sexual transmission	Afghanistan; Democratic Republic of Congo; Guinea; India; Liberia; Sierra Leone; Sudan; Uganda	Thorson *et al*^44^
**Pregnancy**	Long-term economic impact	Zika	Many mothers cannot return to work after childbirth (eg, caregiving roles, complex medical needs of their children, reliance on social welfare payments that have income restrictions)	Brazil	Ambrogi *et al*^95^

CZScongenital Zika syndrome

#### Recovery risk and vulnerability

Sex-mediated risk factors impacting recovery were identified in the context of ebola and Zika. Both viruses have the potential for post-recovery sexual transmission through infected semen, placing sexual partners of recovered males at risk.^26 37 44 46 53 60 71 92^ Pregnancy-specific risks and vulnerabilities during recovery were also observed, including the risk of infant fatality and post-recovery pregnancy/fertility concerns. Maternal death or the inability of an ebola-infected survivor to breastfeed were linked with an increased risk of infant death.^27 81^ Viral persistence of ebola in pregnancy fluids post-maternal recovery posed risks of vertical transmission to fetus/infants and horizontal transmission to caregivers during parturition following maternal recovery.^27 81 88^

#### Access and use of recovery services

Post-outbreak availability and accessibility of services disproportionately impact pregnant women and caregivers of infants/children. Ebola outbreaks have disrupted routine healthcare services, including maternal, child and sexual reproductive health services.^47 62 73^ In the case of Zika, mothers disproportionately bore the burden of caring for children with congenital Zika syndrome (CZS) while facing structural and logistical barriers in accessing medical services and welfare benefits.^46 95^

#### Health-seeking behaviours during recovery

Reduced health-seeking behaviours were found to persist among pregnant women even after ebola outbreaks were declared over due to ongoing stigma and fear of facility-acquired transmission.^56 62 73^

#### Recovery service options

Recovery services, including medical knowledge, medications and specialised care for infants and children with CZS and microcephaly, were found to be lacking.^79 93^

#### Experiences with recovery services

No evidence was identified in the literature pertaining to sex, gender or pregnancy status and experiences with recovery services.

#### Health and social outcomes

Gender-related stigma and its social consequences are evident. Male Ebola survivors experienced stigma due to fears of potential sexual transmission of the virus, attributed to the persistence of Ebola RNA in semen.^44^ Gender disparities and pregnancy status have been found to have differential and overlapping impacts on caregiving responsibilities, long-term economic and educational outcomes, mental health, and social effects. For example, women bear a disproportionate burden as caregivers, even after Zika outbreaks are controlled^40 46 79 95^ and are disproportionately affected by economic loss given their higher likelihood of working in sectors impacted by response measures.^47^ Response efforts are linked to worsening gender gaps in education, as women and girls, who disproportionately shoulder the burden of care or face economic repercussions, encounter increased obstacles in their pursuit of educational opportunities.^7 95^ Mothers caring for children with Zika-related disabilities faced social isolation and stigma,^79 95^ while survivors of Ebola, particularly women, faced heightened stigma and accusations of witchcraft, resulting in psychological trauma and anxiety.^40 71^ Studies examining indirect health outcomes found poorer outcomes for women during Ebola outbreaks and an estimated increase in maternal mortality due to the deaths of healthcare workers from Ebola.^62 72^

### Gaps identified in the literature

Despite advancements in understanding biological susceptibility, disease pathogenesis and treatment responses, a significant lack of sex-disaggregated data persists during outbreaks.^38 47 48 72^ Such data are often not routinely collected^47 48^ or collected late in the outbreak, as observed during the 2014 West Africa Ebola outbreak.^28 38 72^ This hinders the identification of epidemiological differences between women and men. Gaps also exist in sexual transmission data for ebola and Zika, with limited information on transmission risk,^26 92^ routes,^26 92^ infectivity duration in vaginal secretions^25^ and condom effectiveness.^25 44^ The gap in sex-disaggregated data extends to vaccine and treatment safety and efficacy.^65 91^ Even when data are available, comprehending the reasons for observed differences warrants sex and gender-based analysis and further investigations.^31 84^ For instance, more research is needed to understand the drivers of heightened likelihood of hepatitis E virus antibodies in males^31^ and the elevated morbidity and mortality of influenza A (H1N1) in females and pregnant women.^84^

Gendered data and indicators are not systematically collected during outbreaks.^28 40 60 66 72 94^ A review of ebola and Zika outbreaks indicated that less than 1% of published studies reported on gender indicators.^72^ The lack of gender-sensitive targets and indicators in response strategies and monitoring mechanisms contributed to evidence gaps.^28 40 72^ Moreover, the link between gender, attitudes, perceptions and preventive behaviours across diverse cultures during outbreaks remains insufficiently studied, with underlying mechanisms not comprehensively explored.^35 66 74 94^ For example, very few studies examined how gender affects Zika prevention behaviours, and none examined men’s perceptions of the Zika response or their roles in prevention.^74 94^ Research on structural barriers to accessing prevention and healthcare services for different genders is limited but potentially affects disease burden estimates.^35 83^ There is also notable limited research examining the long-term health, social and psychosocial impacts of outbreaks and responses by gender.^28 40 71 76 94^

Not only are differences between men and women infrequently examined, but the literature also underscores gaps in intersectional analyses of how sex, gender and pregnancy intersect with other factors compounding inequities during outbreaks.^38 48 60 72 83^ For instance, gaps in intersectional understanding of Zika risk and outcomes resulted in the most marginalised pregnant women remaining at greatest risk for having children with CZS.^72 83^ Moreover, sociological studies on ebola are limited, impeding understanding of factors driving health outcome disparities.^38 60^

Physiological changes during pregnancy affect susceptibility to diseases, designating pregnant women as a vulnerable group during outbreaks. However, gaps persist in systematically collecting pregnancy data during outbreaks, leading to insufficient insights into epidemiological measures, clinical presentations, outcomes and post-recovery risks.^25 27 30 45 57 61 62 64 67 68 73 81 82 86 93^ For example, due to non-routine reporting of pregnancy status during admissions to ebola treatment centres, outcome indicators for pregnant women cannot be measured.^25 27 62 73 81 82^ There is limited research on impacts of prior ebola infection on subsequent pregnancies or fertility.^25^ Data scarcity also exists on how pregnancy-induced biological changes affect susceptibility and pathogenesis^31 32^ and the impact of prior infections or co-infections on disease in pregnancy.^35 37 86^ Numerous literature called for the critical need for well-powered studies to demonstrate vaccine and treatment safety in pregnant women.^25 27 30 31 35 41 43 45 63 91^ Notably, safety of substances like Zika-recommended mosquito repellents during pregnancy lacks evidence.^63^ Gaps were noted in pregnant women’s experiences in healthcare facilities, such as ebola treatment centres.^73^ One study showed inadequate pregnancy data coupled with gender inequality in healthcare provider decision-making structures led to non-adaptive guidance during ebola outbreaks, affecting maternal deaths.^73^

Evidence gaps also extend to vertical transmission, fetal risks and infant outcomes.^25 30 32 33 36 58 69^ For instance, there are limited insights of the route of potential vertical transmission of dengue virus.^33^ Additionally, there is evidence that limited knowledge of fetal risks from maternal Zika infection^36 69^ affected guidance for pregnant women facing severe fetal outcomes. Variations in Zika-related microcephaly cases across time and geography are unexplained^58^ and limited data about children born to Zika-infected mothers hinders measuring Zika’s lifelong impact.^33 51^

## Discussion

This scoping review demonstrates how sex and gender significantly, yet variably, impact every phase of outbreak preparedness and response. However, it also reveals persistent sex and gender evidence gaps in outbreak response. Additionally, it warns that gender-insensitive policies and programmes in outbreak response reinforce and may exacerbate gender and health inequities.

The available evidence demonstrates that sex and gender as well as pregnancy status significantly influence outbreak and response dynamics throughout all four phases of the outbreak timeline. These disparities encompassed a wide range of risk factors for disease, vulnerability, access to services, health-seeking behaviours, treatment options as well as experiences in health settings and health and social outcomes and consequences. The findings highlight that while sex differences and gender disparities in outbreaks vary by disease, setting and population, they play significant roles in shaping outbreak dynamics and need to be considered from an intersectional perspective.

Despite this recognised significance, the persistent sex and gender evidence gaps in outbreak response are notably demonstrated in the relatively few articles from 2012 to 2022 that considered sex or gender dimensions of disease outbreaks. The majority of the articles that did incorporate sex and/or gender further cited sex and gender evidence gaps as limiting their own analysis or general conclusions.^25^,^2830^ During outbreaks and public health emergencies, collection and analysis of sex- and gender-related data are often overlooked as the focus is on quick and simple actions.^6 18 40 47 72^ For example, gender-sensitive data on access to SRH services or contraception usage are often not collected.^40^ Furthermore, intersectional gender analyses, accounting for compounding effects of other social stratifiers, are frequently overlooked.^3^,^5^

The articles reviewed also highlight evidence that the failure to consider gender dimensions of outbreaks and public health emergencies can reinforce gender inequalities during and after outbreaks, leading to long-term negative health and social outcomes.^6 40 47 74 83 94^ Zika prevention response efforts have been criticised for placing the burden of preventing both pregnancy and infection solely on women, without acknowledging the gender-related barriers they faced.^47 50 72 76 83 94^ Evidence from the COVID-19 pandemic further demonstrates how gender-insensitive policies risk reinforcing gender and other health inequities.^2 17 18 96^

Intersectional gender analyses are critically needed in outbreak and public health emergencies for developing and implementing effective response strategies that account for differential outbreak experiences and promote equitable outcomes. Systematically collecting and reporting sex-disaggregated data, sex-specific data and gender-sensitive indicators, such as those outlined in Gender Responsive Monitoring and Evaluation,^97^ at the local level, routine sex and gender-based analysis, and the use of these in response decision and planning would considerably improve the effectiveness and equitability of response efforts. It would also fill gaps in crucial information for preparedness and evidence-based decisions in future responses^98^ and mitigate the unintended harms that can result from failure to fully consider sex and gender dimensions.

Various toolkits and guidance documents have been devised to improve the consideration of sex and gender aspects during outbreaks. However, much of this guidance tends to be disease-specific or focused in one area of response, such as surveillance. Due to the lack of appropriate guidance, the GOARN Analytics for Outbreaks Working Group called for the adaptation of the widely cited SAGER guidelines for Integrated Outbreak Analytics (SAGER-IOA). Integrated Outbreak Analytics (IOA) is a collaborative, multi-disciplinary and multi-actor approach to better understand outbreak dynamics, risk factors and underlying causes at the local level.^99 100^ SAGER-IOA development focuses on gathering data on sex, age and gender during outbreaks, conducting collaborative and integrated analyses to discern how these factors interact with outbreak responses, and applying this data for swift, evidence-based decision-making. SAGER-IOA will address a critical need by offering a solid framework with flexible guidelines for incorporating sex and gender considerations into outbreak and public health emergency responses to address the sex and gender evidence gap and support gender-responsive actions.

### Limitations

Although our scoping review focused on the inclusion of sex and gender in outbreak responses in LMICs, there is a substantial body of research on sex and gender differences in high-income countries and non-outbreak contexts that may be generalisable to these settings. The exclusion of non-English articles may have also limited the comprehensiveness of our findings. Furthermore, differentiating the dynamics of sex and gender during outbreaks is inherently complex, sex and gender intersect in ways that influence health outcomes, and the underlying drivers are often unclear. Evidence gaps further complicate gender analyses, especially considering that gender itself is a multifaceted construct. This challenge is compounded by the fact that many of the reviewed articles conflate sex and gender. While we aimed for consistency and transparency, the categories of ‘sex’ and ‘gender’ used here are broad qualifiers that rely on how these aspects were interpreted in the reviewed studies.

Although public and patient involvement is critical for research on sex and gender dynamics in outbreaks, the methodological aims and resource limitations of this scoping review made such involvement impractical. This review sought to map and synthesise existing evidence, but future research and follow-up studies should incorporate public and patient perspectives to help address ongoing evidence gaps and identify effective paths forward.

## Conclusion

This scoping review underscores the risks of overlooking sex and gender dimensions in outbreak analytics and response, across prevention, detection, treatment/management and recovery efforts. The evidence unequivocally illustrates that neglecting sex and gender dynamics during outbreaks yields adverse consequences, such as missed information, misguided actions across response phases and worse health outcomes for marginalised groups. Such negligence not only weakens the foundation of current and future outbreak responses but also perpetuates gender inequalities, erodes trust in health systems and contributes to enduring adverse health, social, economic and security ramifications. While a one-size-fits-all approach is untenable for all outbreaks and contexts, a comprehensive, adaptable and actionable strategy that systematically integrates sex and gender dimensions throughout all outbreak phases, grounded in local realities, is urgently required.

## supplementary material

10.1136/bmjgh-2024-015900 online supplemental file 1

10.1136/bmjgh-2024-015900 online supplemental file 2

10.1136/bmjgh-2024-015900 online supplemental file 3

10.1136/bmjgh-2024-015900 online supplemental file 4

10.1136/bmjgh-2024-015900 online supplemental file 5

10.1136/bmjgh-2024-015900 online supplemental file 6

10.1136/bmjgh-2024-015900 online supplemental file 7

## Data Availability

All data relevant to the study are included in the article or uploaded as supplementary information.
