# Supplementary material for: Beyond COVID-19, the case for collecting, analysing and using sex-disaggregated data and gendered data to inform outbreak response: a scoping review
Source: BMJ Glob Health. 2025 Jan 15;10(1):e015900. doi: 10.1136/bmjgh-2024-015900 (PMC11749539; doi:10.1136/bmjgh-2024-015900)
Supplement: online supplemental file 3 [file bmjgh-10-1-s003.pdf]

Supplemental Table A. Database Search Strategy

Database	Strategy	Run Date	Records
**Medline (OVID) 1946-**	((Sex OR gender) ADJ5 (data ADJ5 (collect* OR analy* OR survey* OR aggregat* OR disaggregat*))) OR ((Sex OR gender* OR biological) ADJ5 differen*) OR sex factor* OR sex-based factor* OR gender factor* OR gender-based factor* OR (gender* ADJ2 inequit*) OR (gender* ADJ2 equit*) OR (gender* ADJ2 disparit*) OR (gender* ADJ2 disaggregat*) OR gender analysis OR gender-based analysis OR ((wom?n OR sex* OR gender*) ADJ5 (equity OR disparit* OR unequal OR inequit* OR inequalit* OR vulnerab*))<br>AND<br>(disease outbreak* OR emergency response OR disaster response OR global health OR humanitarian cris?s OR humanitarian emergenc* OR complex emergenc* OR infectious disease* OR influenza OR viral hemorrhagic fever* OR Ebola OR Lassa OR Marburg OR Dengue OR Cholera OR Measles OR Zika OR Polio* OR Yellow Fever OR Malaria OR Hepatitis OR Pneumonic Plague OR MERS OR SARS OR Meningitis OR Tuberculosis OR Diphtheria OR Pertussis)<br>NOT<br>(Exp Animals/ OR (animal* ADJ2 model*)) NOT exp humans/<br>NOT<br>(Exp north america/ OR exp Europe/)<br>Limit 2012 - ; English	04/12/2022	5660
**Embase (OVID) 1974-**	((Sex OR gender) ADJ5 (data ADJ5 (collect* OR analy* OR survey* OR aggregat* OR disaggregat*))) OR ((Sex OR gender* OR biological) ADJ5 differen*) OR sex factor* OR sex-based factor* OR gender factor* OR gender-based factor* OR (gender* ADJ2 inequit*) OR (gender* ADJ2 equit*) OR (gender* ADJ2 disparit*) OR (gender* ADJ2 disaggregat*) OR gender analysis OR gender-based analysis OR ((wom?n OR sex* OR gender*) ADJ5 (equity OR disparit* OR unequal OR inequit* OR inequalit* OR vulnerab*))<br>AND<br>(disease outbreak* OR emergency response OR disaster response OR global health OR humanitarian cris?s OR humanitarian emergenc* OR complex emergenc* OR infectious disease* OR influenza OR viral hemorrhagic fever* OR Ebola OR Lassa OR Marburg OR Dengue OR Cholera OR	04/12/2022	8068<br><br>-2244 duplicates<br><br>=5824 unique items

	Measles OR Zika OR Polio* OR Yellow Fever OR Malaria OR Hepatitis OR Pneumonic Plague OR MERS OR SARS OR Meningitis OR Tuberculosis OR Diphtheria OR Pertussis)<br><br>NOT<br><br>(Exp Animal/ OR (animal* ADJ2 model*)) NOT exp human/<br><br>Limit 2012 - ; English; NOT pubmed/medline; NOT conference abstract.pt		
**Global Health (OVID) 1910-**	((Sex OR gender) ADJ5 (data ADJ5 (collect* OR analy* OR survey* OR aggregat* OR disaggregat*))) OR ((Sex OR gender* OR biological) ADJ5 differen*) OR sex factor* OR sex-based factor* OR gender factor* OR gender-based factor* OR (gender* ADJ2 inequit*) OR (gender* ADJ2 equit*) OR (gender* ADJ2 disparit*) OR (gender* ADJ2 disaggregat*) OR gender analysis OR gender-based analysis OR ((wom?n OR sex* OR gender*) ADJ5 (equity OR disparit* OR unequal OR inequit* OR inequalit* OR vulnerab*))<br><br>AND<br><br>(disease outbreak* OR emergency response OR disaster response OR global health OR humanitarian cris?s OR humanitarian emergenc* OR complex emergenc* OR infectious disease* OR influenza OR viral hemorrhagic fever* OR Ebola OR Lassa OR Marburg OR Dengue OR Cholera OR Measles OR Zika OR Polio* OR Yellow Fever OR Malaria OR Hepatitis OR Pneumonic Plague OR MERS OR SARS OR Meningitis OR Tuberculosis OR Diphtheria OR Pertussis)<br><br>Limit 2012 - ; English	04/12/2022	3500<br><br>-1926 duplicates<br><br>=1574 unique items
**Scopus**	TITLE-ABS-KEY(((Sex OR gender) W/5 (data W/5 (collect* OR analy* OR survey* OR aggregat* OR disaggregat*))) OR ((Sex OR gender* OR biological) W/5 differen*) OR "sex factor*" OR "sex-based factor*" OR "gender factor*" OR "gender-based factor*" OR (gender* W/2 inequit*) OR (gender* W/2 equit*) OR (gender* W/2 disparit*) OR (gender* W/2 disaggregat*) OR "gender analysis" OR "gender-based analysis" OR ((wom?n OR sex* OR gender*) W/5 (equity OR disparit* OR unequal OR inequit* OR inequalit* OR vulnerab*))) AND TITLE-ABS-KEY("disease outbreak*" OR "emergency response" OR "disaster response" OR "global health" OR "humanitarian cris?s" OR "humanitarian emergenc*" OR "complex emergenc*" OR "infectious disease*" OR influenza OR "viral hemorrhagic fever*" OR Ebola OR Lassa	04/12/2022	1898<br><br>-247 duplicates<br><br>=1651 unique items

	OR Marburg OR Dengue OR Cholera OR Measles OR Zika OR Polio* OR "Yellow Fever" OR Malaria OR Hepatitis OR "Pneumonic Plague" OR MERS OR SARS OR COVID OR COVID-19 OR coronavirus* OR Meningitis OR Tuberculosis OR Diphtheria OR Pertussis) AND NOT INDEX(medline) AND NOT INDEX(embase)<br><br>Limit 2012 - ; English		
**Global Index Medicus (WHO)**	"sex factor" OR "sex factors" OR "sex-based factor" OR "sex-based factors" OR "gender factor" OR "gender factors" OR "gender-based factor" OR "gender-based factors" OR (gender AND (inequity OR inequities OR disparity OR disparities)) OR ((gender OR sex) AND (disaggregated OR disaggregation OR aggregated OR aggregation)) OR "gender analysis" OR "gender-based analysis" OR "gender differences" OR "sex-based differences"<br><br>AND<br><br>Outbreak OR outbreaks OR epidemic OR epidemics OR "emergency response" OR "disaster response" OR "emerging infectious disease" OR influenza OR "viral hemorrhagic fever*" OR Ebola OR Lassa OR Marburg OR Dengue OR Cholera OR Measles OR Zika OR Polio* OR "Yellow Fever" OR Malaria OR Hepatitis OR "Pneumonic Plague" OR MERS OR SARS OR Meningitis	04/12/2022	1014<br><br>-404 duplicates<br><br>=618 unique items
