# Supplementary material for: Beyond COVID-19, the case for collecting, analysing and using sex-disaggregated data and gendered data to inform outbreak response: a scoping review
Source: BMJ Glob Health. 2025 Jan 15;10(1):e015900. doi: 10.1136/bmjgh-2024-015900 (PMC11749539; doi:10.1136/bmjgh-2024-015900)
Supplement: online supplemental file 4 [file bmjgh-10-1-s004.pdf]

Supplemental Table B. Evidence of sex, gender, and pregnancy status-related implications for prevention

RESPONSE PHASE: PREVENTION					
Data Categories	Theme	Disease	Evidence	Country	First Author, Year
Prevention risk factors & vulnerability					
Sex	Epidemiological measures of disease	Cholera	Higher prevalence (55-65% of cases) and greater risk for symptomatic cholera among females	Uganda	Cummings, 2012
			Higher prevalence among females above age 5	Countries not listed	Sevilimedu, 2016
		Dengue	Higher prevalence among males aged 15 to 75 and higher risk of severe outcomes among males	Pakistan	Aamir, 2014
			Higher prevalence of suspected dengue among females aged 10-49 compared to females of other ages in the study	Brazil	Feitoza, 2017
		Ebola	Higher incidence among females	Brazil; Colombia; Ecuador; El Salvador; Guinea; Liberia; Sierra Leone	Davies, 2016
				Uganda	Kankya, 2019
			Higher case fatality rate among females	Nigeria	Fawole, 2016
			Risk of death among females increases with vaginal bleeding, bleeding gums or epistaxis	Guinea; Liberia; Sierra Leone; Other	Bebell, 2017
			Higher case fatality rate among males	Democratic Republic of Congo; Gabon; Guinea; Liberia; Republic of Congo; Sierra Leone; Sudan; Uganda	Nkangu, 2017
				Guinea; Liberia; Sierra Leone; South Sudan; Uganda	Gomes, 2017
			Higher case fatality rate among males when adjusting for multiple other factors (pregnancy excluded)	Guinea; Liberia; Sierra Leone; Other	Bebell, 2017
		Hepatitis E	Higher prevalence, severity, and case fatality rate among females	Uganda	Amanya, 2017
			Prevalence of cases among males ranges from 70% (age 25-33; Nigeria) to 2% (age <15; Egypt)	Algeria; Burkina Faso; Burundi; Cameroon; Central African Republic; Chad; Cote d'Ivoire; Democratic Republic of Congo; Djibouti; Eritrea; Ethiopia; Gabon; Ghana; Kenya; Madagascar; Morocco; Namibia; Nigeria; Senegal; Somalia; South Africa; South Sudan; Sudan; Tanzania; Tunisia; Zambia	Kim, 2014
			Higher risk of symptoms and increased antibodies among males	India; Egypt; South Africa; Bangladesh; Other	Kmush, 2015
		Influenza	Higher incidence of H5N1, higher proinflammatory cytokines, and chemokine responses among females	Arab Republic of Egypt; Indonesia; Malaysia; Vietnam; Other	Liu, 2016
			Higher prevalence among males but higher risk of severity and case fatality rate among females	India	Mahendra, 2014

		Zika	Higher incidence among females aged 15 to 65 (sexually active age group)	Brazil	Coelho, 2016
			Higher prevalence among females	Colombia	Forero-Martinez, 2020
			Higher predicted baseline rates of microcephaly with structural brain defects in female infants	Brazil; Dominican Republic	Brady, 2019
	Sexual transmission	Ebola	Infection through sexual transmission	Afghanistan; Democratic Republic of Congo; Guinea; India; Liberia; Sierra Leone; Sudan; Uganda	Thorson, 2016
			Ribonucleic Acid (RNA) in semen for months post-recovery (prolonged potential for sexual transmission)	Guinea; Liberia; Sierra Leone; Other	Bebell, 2017
				Guinea; Liberia; Sierra Leone; Other	Bebell, 2017
				Brazil; Colombia; Ecuador; El Salvador; Guinea; Liberia; Sierra Leone	Davies, 2016
				Guinea; Liberia; Sierra Leone; South Sudan; Uganda	Gomes, 2017
				Afghanistan; Democratic Republic of Congo; Guinea; India; Liberia; Sierra Leone; Sudan; Uganda	Thorson, 2016
			RNA in vaginal secretions (paucity of data on potential infectivity)	Guinea; Liberia; Sierra Leone; Other	Bebell, 2017
				Guinea; Liberia; Sierra Leone; South Sudan; Uganda	Gomes, 2017
				Afghanistan; Democratic Republic of Congo; Guinea; India; Liberia; Sierra Leone; Sudan; Uganda	Thorson, 2016
			Disease may be transmitted sexually by leaking through small pores in latex condoms; handling of used condoms	Afghanistan; Democratic Republic of Congo; Guinea; India; Liberia; Sierra Leone; Sudan; Uganda	Thorson, 2016
		Zika	Infection through sexual transmission	Brazil	Calvet, 2016
				Brazil; Other	Arias, 2020
				Brazil; Colombia	Tambo, 2016
				Colombia	Tirado, 2020
			Sexual transmission spreads virus beyond tropical/ subtropical regions	Brazil	Coelho, 2016
			Higher replicative RNA load in semen samples than urine or blood	Brazil	Calvet, 2016
			Male-to-female sexual transmission higher than male-to-male	Brazil	Coelho, 2016
				Angola; American Samoa; Brazil; Columbia; Cuba; Dominican Republic; Ecuador; Guatemala; Guinea-Bissau; Haiti; Honduras; India; Jamaica; Nicaragua; Panama; Peru; Puerto Rico; Suriname; Thailand; Venezuela; Vietnam	Musso, 2019
			Male-to-female sexual transmission can occur whether the infected male partner is symptomatic or asymptomatic	Angola; American Samoa; Brazil; Columbia; Cuba; Dominican Republic; Ecuador; Guatemala; Guinea-Bissau;	Musso, 2019

				Haiti; Honduras; India; Jamaica; Nicaragua; Panama; Peru; Puerto Rico; Suriname; Thailand; Venezuela; Vietnam	
			RNA in semen post-recovery (prolonged potential for sexual transmission)	Colombia; Cuba; Dominican Republic; El Salvador; Guyana; Haiti; Honduras; Mexico; Nicaragua; Other	Vlassoff, 2018
				Brazil; Other	Arias, 2020
				Ecuador	Casapulla, 2018
				Brazil	Coelho, 2016
				Brazil	Calvet, 2016
			RNA has been detected in semen up to 370 days after onset of illness, but shedding of infective viral particles is rare after 30 days from onset	Angola; American Samoa; Brazil; Columbia; Cuba; Dominican Republic; Ecuador; Guatemala; Guinea-Bissau; Haiti; Honduras; India; Jamaica; Nicaragua; Panama; Peru; Puerto Rico; Suriname; Thailand; Venezuela; Vietnam	Musso, 2019
			Transmission through anal sex	Brazil	Calvet, 2016
			Circumcision is associated with lower risk of sexual transmission	Brazil; Colombia	Tambo, 2016
			RNA in vaginal secretions (prolonged potential for sexual transmission)	Colombia; Cuba; Dominican Republic; El Salvador; Guyana; Haiti; Honduras; Mexico; Nicaragua; Other	Vlassoff, 2018
			Knowledge gaps about sexual transmission (prevention efforts focused on vector control)	Ecuador	Casapulla, 2018
				Brazil	Coutinho, 2021
				Dominican Republic	Gurman, 2020
				Colombia	Forero-Martinez, 2020
				Brazil	Marteleto, 2017
	Vaccination	Yellow Fever	Non-serious adverse events following immunisation were higher among females, while serious adverse events were higher among males	Brazil	Lucena, 2020
			Sex-related disparities in reported adverse events following immunisation were largely among reproductive disorders such as genital bleeding, vaginal haemorrhage and menorrhagia	Democratic Republic of Congo	Nzolo, 2018
**Gender**	Burden of care	Ebola	Women bear caregiving burden in public/private domains, impacted by inadequate health systems through unpaid caregiving and low-income healthcare roles	Guinea; Liberia; Sierra Leone	Harman, 2016
				Brazil; Sierra Leone; Uganda; Other	Smith, 2019
				Brazil; Democratic Republic of Congo	Wenham, 2021
	Exposure	Cholera	Men exposed through fishing occupations and increased time spent outside the house	Countries not listed	Sevilimedu, 2016
			Women exposed through childcare, household chores, cleaning and washing up, and caring for domestic livestock	Countries not listed	Sevilimedu, 2016
			Women exposed to through time spent in over-crowded manyattas and caregiving	Uganda	Cummings, 2012

		Dengue	Men exposed through increased time spent outside the house	Pakistan	Aamir, 2014
		Ebola	Women exposed through formal and informal caregiving	Sierra Leone	Bower, 2016
				Brazil; Colombia; Ecuador; El Salvador; Guinea; Liberia; Sierra Leone	Davies, 2016
				Nigeria	Fawole, 2016
				Uganda	Kankya, 2019
				Democratic Republic of Congo; Gabon; Guinea; Liberia; Republic of Congo; Sierra Leone; Sudan; Uganda	Nkangu, 2017
				Democratic Republic of Congo	Pham, 2022
				Brazil; Sierra Leone; Uganda; Other	Smith, 2019
				Brazil; Democratic Republic of Congo	Wenham, 2021
			Women exposed through food preparation	Democratic Republic of Congo	Pham, 2022
			Women exposed through survival-sex strategies	Brazil; Democratic Republic of Congo	Wenham, 2021
			Women exposed through burial practices	Brazil; Colombia; Ecuador; El Salvador; Guinea; Liberia; Sierra Leone	Davies, 2016
				Nigeria	Fawole, 2016
				Democratic Republic of Congo; Gabon; Guinea; Liberia; Republic of Congo; Sierra Leone; Sudan; Uganda	Nkangu, 2017
				Democratic Republic of Congo	Pham, 2022
			Gender-differences in disease exposure due to burial practices; washing and preparation of bodies done by those of the same gender as deceased	Guinea; Liberia; Sierra Leone	Harman, 2016
			Women exposed through cross-border trading	Democratic Republic of Congo; Gabon; Guinea; Liberia; Republic of Congo; Sierra Leone; Sudan; Uganda	Nkangu, 2017
			Men exposed through game hunting	Uganda	Kankya, 2019
			Men exposed to through caring for livestock	Democratic Republic of Congo; Gabon; Guinea; Liberia; Republic of Congo; Sierra Leone; Sudan; Uganda	Nkangu, 2017
		Hepatitis E	Men exposed through time spent outside the house and increased contact with contaminated water sources	India; Egypt; South Africa; Bangladesh; Other	Kmush, 2015
		Influenza	Women exposed to H5N1 through contact with domesticated poultry and fomites in fertiliser	Arab Republic of Egypt; Indonesia; Malaysia; Vietnam; Other	Liu, 2016
			Increased exposure among men due to gender roles and activities	India	Mahendra, 2014
		Zika	Increased exposure among women due to contact with vectors in the house	Brazil	Coelho, 2016
	Healthcare worker safety	Ebola	Caregivers can perform obstetric interventions safely using appropriate personal protective	Guinea	Baggi, 2014
				Sierra Leone	Oduyebo, 2015

			equipment (PPE) and infection control measures (ICM)		
			Colourless and odourless amniotic fluid is highly infectious and PPE breaches may be challenging to identify	Guinea; Liberia; Sierra Leone; Other	Bebell, 2017
			Midwives delivered infected babies without adequate PPE due to symptom absence	Sierra Leone	Bower, 2016
			Delay between foetal death and delivery, which could potentially lead to horizontal transmission to healthcare workers after maternal recovery	Sierra Leone	Bower, 2016
			Lack of PPE for women caregivers (e.g., midwives)	Sierra Leone	Erland, 2017
				Nigeria	Fawole, 2016
	Individual agency	Ebola	Lack of women's autonomy in sexual and reproductive matters (challenges negotiating condom use)	Afghanistan; Democratic Republic of Congo; Guinea; India; Liberia; Sierra Leone; Sudan; Uganda	Thorson, 2016
		Zika	Lack of women's autonomy in sexual and reproductive matters (victims of non-consensual sex, sexual assault, and intimate partner violence)	Colombia; Cuba; Dominican Republic; El Salvador; Guyana; Haiti; Honduras; Mexico; Nicaragua; Other	Vlassoff, 2018
			Lack of women's autonomy in sexual and reproductive matters (intimate partner violence, challenges negotiating condom use - more prevalent among low SES, less educated women)	Brazil	Coutinho, 2021
	Knowledge & awareness	Cholera	Men had greater knowledge of community education efforts on the cause, mode of transmission and how to prevent cholera provided by health authorities	Nigeria	Adeneye, 2016
		Dengue	Women had greater knowledge of symptoms and prevention due to door-to-door community education	Peru	Elson, 2020
		Ebola	Men had greater knowledge of symptoms, causes, and transmission, which were associated with increases in vaccine acceptance, formal care seeking, and self-protective behaviours	Democratic Republic of Congo	Pham, 2022
			The response did not sufficiently adapt messaging or engagement to include women	Democratic Republic of Congo	Pham, 2022
				Brazil; Democratic Republic of Congo	Wenham, 2021
		Zika	Prevention messaging reinforced gender roles	Brazil	Coutinho, 2021
			The response did not sufficiently adapt messaging or engagement to include men, who often have central roles in decision-making about expenditures and healthcare seeking	Colombia; Cuba; Dominican Republic; El Salvador; Guyana; Haiti; Honduras; Mexico; Nicaragua; Other	Vlassoff, 2018
			Males reported hearing about Zika on the internet	Ecuador	Casapulla, 2018
				Brazil	Borges, 2018

			Lack of knowledge regarding sexual transmission among women	Ecuador	Casapulla, 2018
				Colombia	Tirado, 2020
Pregnancy	Epidemiological measures of disease	Dengue	Increased incidence among pregnant women	Brazil	Nascimento, 2017
			Increased incidence among individuals of reproductive ages, including pregnant women	Indonesia	Mulyana, 2020
			Increased incidence serotype 2 (responsible for most severe forms of disease) among pregnant women	Brazil	do Nascimento Einloft, 2021
			Immune suppression during pregnancy may favour more serious infections (e.g., congenital)	Brazil	Feitoza, 2017
			Increased risk of severe disease among pregnant women	Brazil	Coelho, 2016
				Brazil	Feitoza, 2017
				Indonesia	Mulyana, 2020
				India	Sharma, 2016
			Increased risk of severe disease among pregnant women, primarily in the last trimester; risk increases with age	Brazil	Machado, 2013
			Higher case fatality rate among pregnant women	Brazil	Feitoza, 2017
				Brazil	do Nascimento Einloft, 2021
				Brazil	Machado, 2013
				Indonesia	Mulyana, 2020
				India	Sharma, 2016
			Higher case fatality rate among pregnant women; greatest risk in third trimester	Brazil	Nascimento, 2017
		Ebola	Infected pregnant women may fare worse as a result of altered immune state, expanded blood volume and placental infection; no evidence that pregnant women are more susceptible to infection	Guinea; Liberia; Sierra Leone; Other	Bebell, 2017
			Increased risk of severe disease among pregnant women	Guinea	Baggi, 2014
				Sierra Leone	Bower, 2016
			Higher case fatality rate among pregnant women	Guinea	Baggi, 2014
				Guinea; Liberia; Sierra Leone; Other	Bebell, 2017
				Sierra Leone	Bower, 2016
				Sierra Leone	Erland, 2017
				Sierra Leone	Lyman, 2018
				Democratic Republic of the Congo; Uganda	Muehlenbachs, 2017
				Sierra Leone	Oduyebo, 2015
		Hepatitis E	Increased incidence among pregnant women	India; Egypt; South Africa; Bangladesh; Other	Kmush, 2015
			Higher seroprevalence among pregnant women (varies by country)	Algeria; Burkina Faso; Burundi; Cameroon; Central African Republic; Chad; Cote d'Ivoire; Democratic Republic of Congo; Djibouti; Eritrea; Ethiopia; Gabon; Ghana; Kenya; Madagascar; Morocco; Namibia; Nigeria;	Kim, 2014

				Senegal; Somalia; South Africa; South Sudan; Sudan; Tanzania; Tunisia; Zambia	
			Higher prevalence among pregnant women in third trimester	Pakistan	Tahira, 2013
			Increased risk of severe disease among pregnant women	Uganda	Amanya, 2017
				Pakistan	Khaskheli, 2015
				Algeria; Burkina Faso; Burundi; Cameroon; Central African Republic; Chad; Cote d'Ivoire; Democratic Republic of Congo; Djibouti; Eritrea; Ethiopia; Gabon; Ghana; Kenya; Madagascar; Morocco; Namibia; Nigeria; Senegal; Somalia; South Africa; South Sudan; Sudan; Tanzania; Tunisia; Zambia	Kim, 2014
			Increased risk of severe disease among pregnant women, greatest risk in 2nd and 3rd trimester	Pakistan	Tahira, 2013
			Increased risk of severe disease among pregnant women, greatest risk in 3rd trimester	India; Egypt; South Africa; Bangladesh; Other	Kmush, 2015
			Increased risk of severe disease among pregnant women may be associated with malnutrition, diminished cellular immunity and high levels of steroid hormones during pregnancy	Sudan	Rayis, 2013
			Increased risk of severe disease among pregnant women with greatest risk in 3rd trimester; may be due to due to immune suppression and possible replication of Hepatitis E in the placenta	Pakistan	Tahira, 2013
			Higher case fatality rate among pregnant women	Uganda	Amanya, 2017
				Pakistan	Khaskheli, 2015
				India; Egypt; South Africa; Bangladesh; Other	Kmush, 2015
				Sudan	Rayis, 2013
				Pakistan	Tahira, 2013
			Higher case fatality rate among pregnant women, with greater risk during the 3rd trimester	Algeria; Burkina Faso; Burundi; Cameroon; Central African Republic; Chad; Cote d'Ivoire; Democratic Republic of Congo; Djibouti; Eritrea; Ethiopia; Gabon; Ghana; Kenya; Madagascar; Morocco; Namibia; Nigeria; Senegal; Somalia; South Africa; South Sudan; Sudan; Tanzania; Tunisia; Zambia	Kim, 2014
			Maternal age in addition to hormonal, immunological and environmental factors may increase risk for fatal outcomes	Pakistan	Khaskheli, 2015
		Influenza	Increased susceptibility to H5N1 among pregnant women	Arab Republic of Egypt; Indonesia; Malaysia; Vietnam; Other	Liu, 2016

			Increased susceptibility, especially during 3rd trimester	Argentina; Brazil; South Africa; Turkey; Other	Meijer, 2015
			Increased susceptibility and vulnerability among pregnant women due to physiological changes and the suppression of cellular immunity during pregnancy	Countries not listed	Takeda, 2015
			Increased risk of severe disease in pregnant women due to altered respiratory and immune systems, and increasingly attenuated inflammatory responses; greatest risk in 2nd and 3rd trimesters	Arab Republic of Egypt; Indonesia; Malaysia; Vietnam; Other	Liu, 2016
			Increased risk of severe disease in pregnant women	Argentina; Brazil; South Africa; Turkey; Other	Meijer, 2015
			Higher case fatality rate among pregnant women	India	Mahendra, 2014
				Argentina; Brazil; South Africa; Turkey; Other	Meijer, 2015
			Higher H5N1 case fatality rate among pregnant women due to physical changes during pregnancy	Arab Republic of Egypt; Indonesia; Malaysia; Vietnam; Other	Liu, 2016
		Malaria	Increased risk of severe disease among pregnant women due to decreased immunity; greatest risk in 2nd trimester	Burkina Faso; Gabon; Ghana; India; Kenya; Malawi; Mozambique; Nigeria; Rwanda; Senegal; Solomon Islands; Sudan; Uganda	Uneke, 2012
			Frequent asymptomatic cases among pregnant women in malaria endemic areas due to acquired immunity	Burkina Faso; Gabon; Ghana; India; Kenya; Malawi; Mozambique; Nigeria; Rwanda; Senegal; Solomon Islands; Sudan; Uganda	Uneke, 2012
		Zika	Higher incidence among pregnant women	Dominican Republic	Gurman, 2020
	Adverse Pregnancy Outcomes, Foetal and Neonatal Risk	Dengue	Infection is associated with pregnancy complications (e.g., preeclampsia, haemorrhage)	Brazil	do Nascimento Einloft, 2021
				Indonesia	Mulyana, 2020
				Brazil	Nascimento, 2017
			Infection is associated with increased risk to the foetus (e.g., preterm birth, low birthweight, foetal death, etc.)	Brazil	Feitoza, 2017
				India	Sharma, 2016
			Higher risk of foetal death in 1st trimester; Higher risk of low birthweight and preterm birth in 3rd trimester	Brazil	Feitoza, 2017
			Infection is associated with adverse neonatal outcomes (e.g., asphyxia, neonatal death, etc.)	Brazil	Feitoza, 2017
		Ebola	Infection is associated with high foetal case fatality and high neonatal case fatality (0% survival)	Guinea	Baggi, 2014
				Guinea; Liberia; Sierra Leone; Other	Bebell, 2017
				Sierra Leone	Erland, 2017
				Guinea; Liberia; Sierra Leone; South Sudan; Uganda	Gomes, 2017
				Sierra Leone	Lyman, 2018
				Democratic Republic of the Congo; Uganda	Muehlenbachs, 2017
				Sierra Leone	Oduyebo, 2015

			Infection is associated with increased risk for pregnancy complications (e.g., haemorrhage)	Brazil; Colombia; Ecuador; El Salvador; Guinea; Liberia; Sierra Leone	Davies, 2016
				Guinea; Liberia; Sierra Leone; South Sudan; Uganda	Gomes, 2017
				Democratic Republic of the Congo; Uganda	Muehlenbachs, 2017
		Hepatitis E	Infection is associated with increased risk to the foetus and neonate (e.g., premature rupture of membranes, preterm birth, low birthweight, foetal death, neonatal death)	India; Egypt; South Africa; Bangladesh; Other	Kmush, 2015
				Sudan	Rayis, 2013
				Pakistan	Tahira, 2013
		Influenza	H5N1 is associated with increased risk to the foetuses (e.g., spontaneous abortion, preterm birth, and foetal death)	Arab Republic of Egypt; Indonesia; Malaysia; Vietnam; Other	Liu, 2016
			Infection is associated with increased risk to foetuses and neonates (e.g., preterm birth, stillbirth, foetal death and neonatal ICU admission)	Argentina; Brazil; South Africa; Turkey; Other	Meijer, 2015
		Malaria	Infection is associated with increased risk to the foetuses and neonates (e.g., congenital malaria, preterm birth, intrauterine growth retardation, low birthweight, reduced neonatal anthropometric parameters, foetal anaemia, and foetal and infant death)	Burkina Faso; Gabon; Ghana; India; Kenya; Malawi; Mozambique; Nigeria; Rwanda; Senegal; Solomon Islands; Sudan; Uganda	Uneke, 2012
		Zika	Infection is associated with increased risk of pregnancy complications (e.g., spontaneous abortion, emergency caesarean)	Brazil	Brasil, 2016
				Ecuador	Casapulla, 2018
			Infection is associated with severe congenital abnormalities and neurological complications such as Guillain Barre syndrome	Brazil	Calvet, 2016
			Infection is associated with increased risk to foetuses and neonates (e.g., microcephaly, congenital Zika syndrome (CZS), polydactyly, hypospadias, feet deformities, hydrocephalus, etc.)	Brazil; Dominican Republic	Brady, 2019
				Brazil	Brasil, 2016
				Ecuador	Casapulla, 2018
				Brazil; Colombia; Ecuador; El Salvador; Guinea; Liberia; Sierra Leone	Davies, 2016
				Brazil	de Oliveira, 2017
				Colombia	Forero-Martinez, 2020
				Dominican Republic	Gurman, 2020
				Brazil; El Salvador; Other	Johnson, 2017
				Brazil; Other	Linde-Arias, 2020
				Brazil	Marteleto, 2017
				Brazil	Martines, 2016
				Brazil	Meneses, 2017
				Angola; American Samoa; Brazil; Columbia; Cuba; Dominican Republic; Ecuador; Guatemala; Guinea-Bissau; Haiti; Honduras; India; Jamaica; Nicaragua; Panama;	Musso, 2019

				Peru; Puerto Rico; Suriname; Thailand; Venezuela; Vietnam	
				Columbia	Tirado, 2020
				Brazil; Colombia; El Salvador	Wenham, 2021 (Analysing the intersection...)
			Infection is associated with adverse foetal outcomes across all trimesters	Brazil	Brasil, 2016
			Infection in the 1st or 2nd trimester is associated with elevated risk of microcephaly	Brazil; Other	Arias, 2020
				Brazil; Dominican Republic	Brady, 2019
				Angola; American Samoa; Brazil; Columbia; Cuba; Dominican Republic; Ecuador; Guatemala; Guinea-Bissau; Haiti; Honduras; India; Jamaica; Nicaragua; Panama; Peru; Puerto Rico; Suriname; Thailand; Venezuela; Vietnam	Musso, 2019
				Brazil; Colombia; El Salvador	Wenham, 2021 (Analysing the intersection...)
				Brazil	Sousa, 2018
			No association found between infection in 3rd trimester and microcephaly; Infection up to 10 weeks preconception may confer elevated risk	Brazil; Dominican Republic	Brady, 2019
			Infection in the 3rd trimester associated with lower risk of microcephaly since foetus is already formed	Brazil	Sousa, 2018
			Placenta is more sensitive to the virus during the first trimester	Brazil	Mota, 2018
			Infants of mothers aged <18 had significantly lower High-Concentration Zika (HCZ) score when compared with infants of older mothers	Brazil	Meneses, 2017
			Infection is associated with increased foetal and neonatal case fatality rates	Angola; American Samoa; Brazil; Columbia; Cuba; Dominican Republic; Ecuador; Guatemala; Guinea-Bissau; Haiti; Honduras; India; Jamaica; Nicaragua; Panama; Peru; Puerto Rico; Suriname; Thailand; Venezuela; Vietnam	Musso, 2019
				Brazil	Brasil, 2016
				Brazil	Calvet, 2016
				Brazil; Colombia	Tambo, 2016
			Infection is associated with neonatal ICU admissions	Brazil	Brasil, 2016
				Brazil	Meneses, 2017
	Vertical transmission	Dengue	Infection through vertical transmission	India	Sharma, 2016
				Brazil	Feitoza, 2017
				Indonesia	Mulyana, 2020
			Vertical transmission routes (e.g., transplacental, breast milk, intrapartum, or close contact with newborn) have not been well established	Brazil; Other	Marrs, 2016

			Presence of RNA in breast milk	Brazil; Other	Marrs, 2016
		Ebola	Vertical transmission (e.g., placenta, delivery, breast feeding)	Guinea	Baggi, 2014
				Guinea; Liberia; Sierra Leone; Other	Bebell, 2017
				Guinea; Liberia; Sierra Leone; South Sudan; Uganda	Gomes, 2017
				Democratic Republic of the Congo; Uganda	Muehlenbachs, 2017
				Sierra Leone	Oduyebo, 2015
		Hepatitis E	Infection through vertical transmission	Algeria; Burkina Faso; Burundi; Cameroon; Central African Republic; Chad; Cote d'Ivoire; Democratic Republic of Congo; Djibouti; Eritrea; Ethiopia; Gabon; Ghana; Kenya; Madagascar; Morocco; Namibia; Nigeria; Senegal; Somalia; South Africa; South Sudan; Sudan; Tanzania; Tunisia; Zambia	Kim, 2014
				India; Egypt; South Africa; Bangladesh; Other	Kmush, 2015
				Pakistan	Tahira, 2013
		Influenza	Highly pathogenic strains of the virus, such as H5N1, are more likely to be transmitted across the placenta.	Arab Republic of Egypt; Indonesia; Malaysia; Vietnam; Other	Liu, 2016
		Malaria	Transplacental transmission of P. falciparum (i.e., congenital malaria)	Burkina Faso; Gabon; Ghana; India; Kenya; Malawi; Mozambique; Nigeria; Rwanda; Senegal; Solomon Islands; Sudan; Uganda	Uneke, 2012
		Zika	Infection through vertical transmission	Brazil	Calvet, 2016
				Ecuador	Casapulla, 2018
				Brazil	Coutinho, 2021
				Colombia	Forero-Martinez, 2020
				Brazil; Other	Marrs, 2016
				Brazil	Marteleto, 2017
				Brazil	Melo, 2019
				Angola; American Samoa; Brazil; Columbia; Cuba; Dominican Republic; Ecuador; Guatemala; Guinea-Bissau; Haiti; Honduras; India; Jamaica; Nicaragua; Panama; Peru; Puerto Rico; Suriname; Thailand; Venezuela; Vietnam	Musso, 2019
				Brazil	Sousa, 2018
				Brazil; Colombia	Tambo, 2016
				Colombia; Cuba; Dominican Republic; El Salvador; Guyana; Haiti; Honduras; Mexico; Nicaragua; Other	Vlassoff, 2018
			Presence of RNA in breast milk	Ecuador	Casapulla, 2018
				Brazil; Other	Marrs, 2016
			Potential transmission through breastfeeding	Brazil; Colombia	Tambo, 2016
		Zika		Brazil	Sousa, 2018

	Knowledge & awareness		Lack of knowledge among pregnant women (e.g., symptoms, risks, sexual transmission, prevention options)	Colombia	Tirado, 2020
				Colombia; Cuba; Dominican Republic; El Salvador; Guyana; Haiti; Honduras; Mexico; Nicaragua; Other	Vlassoff, 2018
			Lack of knowledge about safe abortion options related to infection among pregnant women	Colombia	Forero-Martinez, 2020
	Individual agency	Zika	Limited autonomy for women regarding family planning and pregnancy; high rates of unintended pregnancies	Brazil; El Salvador; Other	Borges, 2018
				Brazil; Colombia; Ecuador; El Salvador; Guinea; Liberia; Sierra Leone	Davies, 2016
				Brazil	Johnson, 2017
				Brazil; Colombia; Ecuador; El Salvador; Guinea; Liberia; Sierra Leone	Marteleto, 2017
				Brazil	Vlassoff, 2018
	Intersectionality of gender, race/ethnicity, age, socioeconomic status, etc.	Dengue	Low education, young age, living in a peri-urban area associated with infection during pregnancy; Indigenous women higher risk of infection during pregnancy	Brazil	do Nascimento Einloft, 2021
		Hepatitis E	Low socioeconomic status (SES) with limited access to sanitation associated with infection during pregnancy	Pakistan	Khaskheli, 2015
		Zika	Live births with microcephaly were higher among women w/ low SES	Brazil; Other	Arias, 2020
			Limited autonomy for women regarding family planning and pregnancy; high rates of unintended pregnancies	Dominican Republic	Gurman, 2020
			Women with low SES were impacted the most	Brazil; Other	Linde-Arias, 2020
			Low SES associated with more limited autonomy in sexual and reproductive matters	Brazil	Marteleto, 2017
**Access & use of prevention services**					
**Sex**	No evidence identified in the literature				
**Gender**	Availability	Ebola	Lack of PPE for women	Nigeria	Fawole, 2016
		Zika	Limited contraception availability	Colombia; Cuba; Dominican Republic; El Salvador; Guyana; Haiti; Honduras; Mexico; Nicaragua; Other	Vlassoff, 2018
	Accessibility	Cholera	Less access to sanitation and toilets among women; increased open defecation	Countries not listed	Sevilimedu, 2016
		Ebola	Gaps in condom use, especially for youths, commercial sex workers and men who have sex with men	Afghanistan; Democratic Republic of Congo; Guinea; India; Liberia; Sierra Leone; Sudan; Uganda	Thorson, 2016
		Zika	Transportation barriers to access free female and male condoms	Ecuador	Casapulla, 2018
			Women of lower SES have less access to clean water, sanitation, medical care, sexual and reproductive health (SRH)	Brazil	Coutinho, 2021
	Acceptability	Zika	Acceptability of condom use impacted by religious beliefs	Ecuador	Casapulla, 2018

			Gender norms and gender inequalities limit access to SRH services	Colombia	Tirado, 2020
**Pregnancy**	Availability	Zika	Restrictive policies related to SRH and abortions in some countries with outbreaks	Brazil	Coutinho, 2021
				Brazil; Other	Linde-Arias, 2020
				Brazil	Marteleto, 2017
				Brazil	Mota, 2018
				Colombia	Tirado, 2020
			Inadequate contraception options available	Brazil	Borges, 2018
			Increased demand and use of legal and clandestine abortions	Brazil; El Salvador; Other	Johnson, 2017
				Brazil; Colombia; El Salvador	Wenham, 2021 (Analysing the intersection...)
				Brazil	Marteleto, 2020
	Accessibility	Zika	Geographic disparities in access to SRH	Brazil; Colombia; Ecuador; El Salvador; Guinea; Liberia; Sierra Leone	Davies, 2016
				Brazil	Marteleto, 2017
				Colombia	Tirado, 2020
				Brazil; Colombia	Tambo, 2016
				Colombia	Tirado, 2020
				Brazil; Colombia; El Salvador	Wenham, 2021 (Analysing the intersection...)
				Colombia; Cuba; Dominican Republic; El Salvador; Guyana; Haiti; Honduras; Mexico; Nicaragua; Other	Vlassoff, 2018
			Lack of access to contraceptive methods	Brazil; Sierra Leone; Uganda; Other	Smith, 2019
				Dominican Republic	Gurman, 2020
				Brazil	Mota, 2018
				Colombia; Cuba; Dominican Republic; El Salvador; Guyana; Haiti; Honduras; Mexico; Nicaragua; Other	Vlassoff, 2018
			Lack of access to prenatal visits for men due to long wait times and working hours	Dominican Republic	Gurman, 2020
	Affordability	Zika	SES disparities in access to safe abortion in countries with restrictive abortion laws	Brazil; Colombia; Ecuador; El Salvador; Guinea; Liberia; Sierra Leone	Davies, 2016
				Brazil	Marteleto, 2017
				Brazil	Mota, 2018
			SES disparities in prevention methods (e.g., contraception, abortion, avoidance of exposure during pregnancy, repellents, nets)	Brazil	Marteleto, 2017
				Brazil; Sierra Leone; Uganda; Other	Smith, 2019
				Colombia	Tirado, 2020
				Brazil; Colombia; El Salvador	Wenham, 2021 (Analysing the intersection...)
				Brazil	Melo, 2019
	Acceptability	Zika	Religious and social norms shape acceptability of contraception and abortion services	Brazil; Other	Siqueira, 2019
				Colombia; Cuba; Dominican Republic; El Salvador;	Vlassoff, 2018

				Guyana; Haiti; Honduras; Mexico; Nicaragua; Other	
				Brazil; Colombia; El Salvador	Wenham, 2021 (Analysing the intersection...)
			Perception that quality of care is low, or clinics are crowded may impact use of services	Brazil	Marteleto, 2017
**Prevention & prevention-seeking behaviours**					
**Sex**	No evidence identified in the literature				
**Gender**	Decision-making	Zika	Women were more likely to agree that family and friends influenced decision to take action to prevent infection	Ecuador	Casapulla, 2018
			Gender inequalities in decision-making - approximately 48% of women work in the informal sector, only half of whom report being in control of their income	Dominican Republic	Gurman, 2020
	Responsibility	Zika	Gender affects the acceptance and participation in prevention and control programs; Gender norms and SES shape who is responsible for prevention and vector-control activities (i.e., household chores, cleaning, trash removal)	Brazil	Coutinho, 2021
				Dominican Republic	Gurman, 2020
			Public health systems placed responsibility for preventing Zika onto women who had limited ability to prevent transmission	Brazil; Other	Arias, 2020
				Brazil; Other	Linde-Arias, 2020
			Low involvement in prevention measures among men	Brazil	Coutinho, 2021
	Risk perception	Ebola	Women reported engaging in a greater number of self-protective behaviours, perhaps as a result of greater perceived risk of exposure	Democratic Republic of Congo	Pham, 2022
**Pregnancy**	Risk perception	Zika	Increased risk perception in those intending to become pregnant	Brazil	Marteleto, 2017
			Increased risk perception associated with increased prevention behaviours	Brazil	Melo, 2019
			Lower risk perceptions due to unintended pregnancies may reduce prevention behaviours	Brazil	Coutinho, 2021
	Reproductive intentions	Zika	Outbreak influenced pregnancy intentions	Brazil	Borges, 2018
				Brazil	Marteleto, 2017
			Women over 35 were less likely to report that Zika influenced pregnancy intention	Brazil	Borges, 2018
	Practicality	Zika	Impracticality of wearing full coverage clothing reported among pregnant women	Brazil	Sousa, 2018
				Colombia	Tirado, 2020
			Foetal concerns regarding insect repellent-related side effects during pregnancy	Brazil	Melo, 2019
	Sociocultural norms	Zika	Condom use or abstinence during pregnancy is linked to infidelity/trust	Dominican Republic	Gurman, 2020
			Many cultural or religious norms prohibit or discourage open	Colombia; Cuba; Dominican Republic; El Salvador;	Vlassoff, 2018

			discussion of sexual issues among couples and even with health educators	Guyana; Haiti; Honduras; Mexico; Nicaragua; Other	
			Personal beliefs inform pregnant women use of contraception and abortion services	Colombia	Forero-Martinez, 2020
				Colombia; Cuba; Dominican Republic; El Salvador; Guyana; Haiti; Honduras; Mexico; Nicaragua; Other	Vlassoff, 2018
			Men often take on roles/ responsibilities in the house when their partner is in late pregnancy	Dominican Republic	Gurman, 2020
			Abortion decisions are highly dependent upon gender relations in the household and society	Colombia; Cuba; Dominican Republic; El Salvador; Guyana; Haiti; Honduras; Mexico; Nicaragua; Other	Vlassoff, 2018
	Vaccination	Influenza	Lack of evidence on safety of vaccine in pregnant women drove low uptake	Countries not listed	Takeda, 2015
**Prevention Options**					
**Sex**	No evidence identified in the literature				
**Gender**	Gender targeted response efforts	Ebola	Lack of prevention options targeted at safe hunting practices (greater impact on men)	Democratic Republic of Congo; Gabon; Guinea; Liberia; Republic of Congo; Sierra Leone; Sudan; Uganda	Nkangu, 2017
		Ebola	Lack of prevention options targeted at safe informal caregiving practices (greater impact on women)	Democratic Republic of Congo; Gabon; Guinea; Liberia; Republic of Congo; Sierra Leone; Sudan; Uganda	Nkangu, 2017
		Zika	Preventing sexually transmitted diseases required abstinence or protected intercourse for 2 months for infected females and 3 months for infected males	Angola; American Samoa; Brazil; Columbia; Cuba; Dominican Republic; Ecuador; Guatemala; Guinea-Bissau; Haiti; Honduras; India; Jamaica; Nicaragua; Panama; Peru; Puerto Rico; Suriname; Thailand; Venezuela; Vietnam	Musso, 2019
**Pregnancy**	Vaccination	Ebola	Safety and efficacy of vaccine not yet established in pregnant women resulting in limited prevention options	Guinea; Liberia; Sierra Leone; Other	Bebell, 2017
			Pregnant women not eligible for vaccine	Guinea; Liberia; Sierra Leone; South Sudan; Uganda	Gomes, 2017
			Pregnant and breast-feeding women originally not eligible for vaccine; then there was a policy reversal	Democratic Republic of Congo	Pham, 2022
		Hepatitis E	Vaccine is safe for both mother and foetus	Pakistan	Khaskheli, 2015
			Pregnant women not eligible for vaccination; inadvertent vaccination of pregnant women demonstrated that it was safe	India; Egypt; South Africa; Bangladesh; Other	Kmush, 2015
		Influenza	Vaccination of pregnant women is safe and reduces maternal and neonatal morbidity; immunity conferred to infant through placenta or breast milk	Argentina; Brazil; South Africa; Turkey; Other	Meijer, 2015
				Unknown	Takeda, 2015
		Yellow Fever	Pregnant women identified as a vulnerable population with	Democratic Republic of Congo	Nzolo, 2018

			enhanced risk of serious adverse events following vaccination; Women inadvertently given vaccine suffered genital haemorrhage which resulted in a miscarriage		
			Increased risk of spontaneous abortion after vaccination	Brazil; Cameroon; Cote d'Ivoire; Ghana; Mali; Nigeria; Peru; Other	Thomas, 2012
			Evidence of vaccine safety: vaccination adverse event severity unrelated to pregnancy	Brazil	Lucena, 2020
			Evidence of vaccine safety: no severe adverse events from vaccine in pregnant women; no impact on rates of miscarriage, malformations, foetal case fatality, preterm delivery	Brazil; Cameroon; Cote d'Ivoire; Ghana; Mali; Nigeria; Peru; Other	Thomas, 2012
			Pregnant women at high risk of exposure to infection eligible for vaccination	Brazil	Lucena, 2020
			WHO does not recommend vaccination for pregnant and breastfeeding women unless during epidemics or unavoidable travel to a high-risk area	Brazil; Cameroon; Cote d'Ivoire; Ghana; Mali; Nigeria; Peru; Other	Thomas, 2012
	Restrictions	Zika	Global responses to Zika identified pregnancy termination as a prevention measure, but most highly impacted countries had restrictive abortion policies	Brazil	Borges, 2018
				Brazil; Other	Siqueira, 2019
			CZS is usually identified in 2nd or 3rd trimester, when there are increased legal and technical barriers for access to safe abortion services	Brazil; El Salvador; Other	Johnson, 2017
				Brazil	Mota, 2018
				Brazil; Colombia; El Salvador	Wenham, 2021 (Analysing the intersection...)
			Abortions may be permitted in Brazil in cases where CZS put the woman's life at risk	Brazil; Colombia; El Salvador	Wenham, 2021 (Analysing the intersection...)
	Unacceptable prevention options	Zika	Response recommendations to abstain from/postpone pregnancy ignored the fact that in many of the highest risk regions over half of pregnancies were unintended (due to gender inequalities resulting in a lack of autonomy over sexual and reproductive matters and limited access to contraception)	Brazil; El Salvador; Other	Johnson, 2017
				Brazil; Other	Marrs, 2016
				Brazil; Democratic Republic of Congo	Wenham, 2021
**Experiences in health care**					
**Sex**	No evidence identified in the literature				
**Gender**	Information	Zika	Insufficient information provided by public health officials	Brazil; Other	Linde-Arias, 2020
	Engagement	Zika	Lack of engagement among women in health promotion and decision making, emergency committees created failed to include the Ministries of Women or any SRH constituencies	Brazil; Colombia; El Salvador	Wenham, 2021 (Analysing the intersection...)
	Trust	Zika	Women did not trust medical community's capability to tackle	Brazil; Other	Arias, 2020

			virus due to the lack of agreement among medical providers		
**Pregnancy**	Information	Ebola	Lack of communication about the policy reversal around first excluding then allowing pregnant and breast-feeding women to access vaccines created misunderstanding and suspicion	Democratic Republic of Congo	Pham, 2022
		Zika	Inadequate information provided about prevention options (e.g., prevention of sexual transmission, abortion options)	Brazil	Coutinho, 2021
				Brazil	Sousa, 2018
			Intentional omission of information about prevention options by healthcare staff (e.g., emergency contraception, abortion)	Colombia	Forero-Martinez, 2020
				Brazil	Marteleto, 2017
	Targeted advice	Zika	Women of reproductive age were advised to avoid pregnancy	Brazil; Other	Arias, 2020
			Women under 35 were more likely to be advised to avoid pregnancy	Brazil	Borges, 2018
			High SES women reported that healthcare providers advised prevention measures (e.g., insect repellent, avoid high risk areas, etc.)	Brazil	Marteleto, 2017
	Exclusion	Zika	Men feel excluded from reproductive decision-making in hospitals and excluded from attending prenatal visits	Dominican Republic	Gurman, 2020
	Stigma	Zika	Low SES women reported feeling stigmatised when they went to public clinics for contraceptives, oftentimes because of a violation of privacy by staff in health clinics.	Brazil	Marteleto, 2017
	Decision-making	Zika	Abortion decisions are linked to positive or negative interactions with health staff	Colombia; Cuba; Dominican Republic; El Salvador; Guyana; Haiti; Honduras; Mexico; Nicaragua; Other	Vlassoff, 2018
**Health & social outcomes & consequences**					
**Sex**	No evidence identified in the literature				
**Gender**	Indirect consequences	Ebola	Greater secondary effects of response measures on women; increased domestic care responsibilities if schools are closed; increasing rates of gender-based violence	Brazil; Democratic Republic of Congo	Wenham, 2021
**Pregnancy**	Maternal morbidity and mortality	Zika	Misalignment of response plans with sexual and reproductive policies, resulted in spikes in clandestine and unsafe abortions with associated maternal mortality	Brazil	Borges, 2018
				Colombia	Forero-Martinez, 2020
				Brazil	Mota, 2018
				Brazil; Colombia; Ecuador; El Salvador; Guinea; Liberia; Sierra Leone	Davies, 2016
			Low SES associated with greater chance of unsafe abortions	Brazil	Marteleto, 2017
	Population	Zika	Outbreak in Brazil was associated with a decrease of more than 100,000 births between September 2015 and December	Angola; American Samoa; Brazil; Columbia; Cuba; Dominican Republic; Ecuador; Guatemala; Guinea-Bissau;	Musso, 2019

			2016, which may have resulted from postponement of pregnancies and an increase in clandestine abortions.	Haiti; Honduras; India; Jamaica; Nicaragua; Panama; Peru; Puerto Rico; Suriname; Thailand; Venezuela; Vietnam	
