# Supplementary material for: Beyond COVID-19, the case for collecting, analysing and using sex-disaggregated data and gendered data to inform outbreak response: a scoping review
Source: BMJ Glob Health. 2025 Jan 15;10(1):e015900. doi: 10.1136/bmjgh-2024-015900 (PMC11749539; doi:10.1136/bmjgh-2024-015900)
Supplement: online supplemental file 5 [file bmjgh-10-1-s005.pdf]

Supplemental Table C. Evidence of sex, gender, and pregnancy status-related implications for detection

Data Category	Theme	Disease	Evidence	Country	First Author, Year
**Detection risk & vulnerability**					
**Sex**	No evidence identified in the literature				
**Gender**	No evidence identified in the literature				
**Pregnancy**	Case definition	Ebola	Pregnant women did not meet case definition but tested positive	Sierra Leone	Bower, 2016
			Case definition including bleeding and fever led to high rates of suspected cases in pregnant women that were later found to be negative	Sierra Leone	Erland, 2017
			Not all infected pregnant women will develop hemorrhagic symptoms	Sierra Leone	Oduyebo, 2015
		Zika	Case definition including presence of fever would miss over 70% of cases	Brazil	Brasil, 2016
			Broad case definition identified adverse outcomes in 42% of exposed foetuses and infants, but microcephaly cases were overestimated due to nonspecific case definition, detected in only 55% of initially reported cases	Angola; American Samoa; Brazil; Columbia; Cuba; Dominican Republic; Ecuador; Guatemala; Guinea-Bissau; Haiti; Honduras; India; Jamaica; Nicaragua; Panama; Peru; Puerto Rico; Suriname; Thailand; Venezuela; Vietnam	Musso, 2019
	Pregnancy masking symptoms	Dengue	Clinical manifestations may resemble pregnancy-related conditions (hemoconcentration & hemolysis elevated liver enzymes low platelet count (HELLP) syndrome), complicating detection for physicians	Brazil	do Nascimento Einloft, 2021
				Indonesia	Mulyana, 2020
				India	Sharma, 2016
			Underestimation of Dengue hemorrhagic fever due to compromised identification of plasma leakage syndrome during gestational weeks 7-32, due to increased intravascular volume	Brazil	Machado, 2013
		Ebola	Pregnancy can affect the presentation, prognosis and complicate detection	Sierra Leone	Bower, 2016
				Sierra Leone	Erland, 2017
			2014-2015 West Africa outbreak data estimates < 1.5% of pregnant women with hemorrhagic or febrile symptoms will have confirmed infection due to late pregnancy symptoms mimicking Ebola	Guinea; Liberia; Sierra Leone; Other	Bebell, 2017
**Access & use of screening/diagnostic services**					
**Sex**	No evidence identified in the literature				
**Gender**	No evidence identified in the literature				
**Pregnancy**	Availability	Zika	Limited testing and screening capacity in resource-poor settings may hinder detection of infection/exposed pregnant women, foetuses and infants	Brazil	Ambrogi, 2021
				Brazil; Dominican Republic	Brady, 2019
				Angola; American Samoa; Brazil; Columbia; Cuba; Dominican Republic; Ecuador; Guatemala; Guinea-Bissau; Haiti; Honduras; India; Jamaica; Nicaragua; Panama; Peru; Puerto Rico; Suriname; Thailand; Venezuela; Vietnam	Musso, 2019
**Diagnostic-seeking behaviours**					
**Sex**	No evidence identified in the literature				
**Gender**	Detection bias	Dengue	Increased detection and reporting among reproductive-age women	Brazil	Coelho, 2016

			Decreased detection and reporting among Asian women due to care-seeking from traditional practitioners	Pakistan	Aamir, 2014
		Zika	Increased detection among reproductive-age women due to greater healthcare use (more likely to seek care due to pregnancy concerns)	Brazil	Coelho, 2016
**Pregnancy**	Detection bias	Dengue	Higher incidence among pregnant women may be due to increased use of formal healthcare services and prioritised screening	Brazil	Nascimento, 2017
		Ebola	Underestimation of infected pregnant women due to presenting later for healthcare in resource-limited settings	Guinea; Liberia; Sierra Leone; Other	Bebell, 2017
		Zika	Higher incidence among pregnant women may be due to increased use of formal healthcare services and prioritised screening (microcephaly/foetal concerns)	Brazil	Coelho, 2016
				Brazil	de Oliveira, 2017
				Dominican Republic	Gurman, 2020
**Screening & diagnostic options**					
**Sex**	RNA detection	Ebola	RNA detected in semen up to 284 days after symptom onset	Guinea; Liberia; Sierra Leone; Other	Bebell, 2017
				Brazil; Colombia; Ecuador; El Salvador; Guinea; Liberia; Sierra Leone	Davies, 2016
				Guinea; Liberia; Sierra Leone; South Sudan; Uganda	Gomes, 2017
				Afghanistan; Democratic Republic of Congo; Guinea; India; Liberia; Sierra Leone; Sudan; Uganda	Thorson, 2016
			RNA detected in semen post-recovery	Guinea; Liberia; Sierra Leone; Other	Bebell, 2017
			RNA detected in vaginal fluids/secretions up to 33 days after symptom onset	Guinea; Liberia; Sierra Leone; Other	Bebell, 2017
				Guinea; Liberia; Sierra Leone; South Sudan; Uganda	Gomes, 2017
				Afghanistan; Democratic Republic of Congo; Guinea; India; Liberia; Sierra Leone; Sudan; Uganda	Thorson, 2016
		Zika	Semen has higher levels of RNA compared to urine or blood	Brazil	Calvet, 2016
			RNA detected in semen after clearance from blood and urine	Brazil; Other	Marrs, 2016
				Colombia; Cuba; Dominican Republic; El Salvador; Guyana; Haiti; Honduras; Mexico; Nicaragua; Other	Vlassoff, 2018
			RNA detected in vaginal secretions after clearance from blood and urine		
			RNA detected in semen up to 370 days after onset of symptoms	Brazil; Other	Arias, 2020
				Brazil	Calvet, 2016
				Ecuador	Casapulla, 2018
				Brazil	Coelho, 2016
				Brazil; Other	Linde-Arias, 2020
				Angola; American Samoa; Brazil; Columbia; Cuba; Dominican Republic; Ecuador; Guatemala; Guinea-Bissau; Haiti; Honduras; India; Jamaica; Nicaragua; Panama; Peru; Puerto Rico; Suriname; Thailand; Venezuela; Vietnam	Musso, 2019

**Gender**	No evidence identified in the literature				
**Pregnancy**	Absence of diagnostic testing	Dengue	Routine neonatal diagnostic testing (PCR) is not conducted	Indonesia	Mulyana, 2020
				India	Sharma, 2016
		Ebola	Routine foetal and neonatal diagnostic testing are not conducted	Guinea	Baggi, 2014
				Guinea; Liberia; Sierra Leone; Other	Bebell, 2017
				Sierra Leone	Lyman, 2018
			Comprehensive diagnostic testing to confirm maternal death is not conducted	Guinea; Liberia; Sierra Leone; Other	Bebell, 2017
				Sierra Leone	Lyman, 2018
	Diagnostic challenges	Zika	Diagnostic testing for Zika and congenital Zika infection are sub-optimal	Brazil; Dominican Republic	Brady, 2019
				Angola; American Samoa; Brazil; Columbia; Cuba; Dominican Republic; Ecuador; Guatemala; Guinea-Bissau; Haiti; Honduras; India; Jamaica; Nicaragua; Panama; Peru; Puerto Rico; Suriname; Thailand; Venezuela; Vietnam	Musso, 2019
			Diagnosing microcephaly prenatally is challenging due to head circumference variations, measurement interpretation limitations, and inconsistent definitions in the literature	Brazil; Other	Marrs, 2016
			Serological testing for can be unreliable due to cross-reactivity with Dengue, Chikungunya, and Yellow Fever	Brazil; Other	Marrs, 2016
				Colombia; Cuba; Dominican Republic; El Salvador; Guyana; Haiti; Honduras; Mexico; Nicaragua; Other	Vlassoff, 2018
			Exposure effects, including developmental abnormalities (e.g., microcephaly, seizures, hearing loss, visual impairment, dysphagia and more), may not be detectable until the second trimester of pregnancy or up to 12 months after birth, with delayed manifestations being possible	Brazil; Dominican Republic	Brady, 2019
				Brazil	Brasil, 2016
				Brazil	de Oliveira, 2017
				Brazil	Meneses, 2017
				Angola; American Samoa; Brazil; Columbia; Cuba; Dominican Republic; Ecuador; Guatemala; Guinea-Bissau; Haiti; Honduras; India; Jamaica; Nicaragua; Panama; Peru; Puerto Rico; Suriname; Thailand; Venezuela; Vietnam	Musso, 2019
				Brazil; Colombia; El Salvador	Wenham, 2021
	RNA detection	Ebola	RNA detected in samples of recovered mothers (vaginal secretions, placenta, amniotic fluid, umbilical cord, and neonatal buccal swab), despite negative blood tests	Guinea	Baggi, 2014
				Sierra Leone	Oduyebo, 2015
			RNA detected in vaginal secretions and breast milk	Guinea; Liberia; Sierra Leone; South Sudan; Uganda	Gomes, 2017
**Experiences with diagnostics**					
**Sex**	No evidence identified in the literature				
**Gender**	No evidence identified in the literature				
**Pregnancy**	Delayed and/or Misdiagnosis	Zika	Delayed CZS diagnosis in newborns; 76% of suspected cases in Alagoas from 2015-2016 were either discarded or pending final diagnosis	Brazil	Ambrogi, 2021

			Prolonged wait times for exams		
			Delayed or no diagnosis	Brazil; Dominican Republic	Brady, 2019
			Cases resemble Chikungunya and Dengue which resulted in misdiagnosis or delayed/ambiguous diagnosis	Colombia	Tirado, 2020
			~60% of infants diagnosed with microcephaly prenatally by ultrasound are found to have pathological microcephaly at birth	Brazil; Other	Marrs, 2016
	Lack of provider support	Zika	Pregnant women reported a lack of support, guidance and information about Zika from healthcare staff	Colombia	Tirado, 2020
**Health & social outcomes & consequences**					
**Sex**	No evidence identified in the literature				
**Gender**	No evidence identified in the literature				
**Pregnancy**	Blood transfusion risk	Zika	No licensed blood donor screening tests	Brazil; Other	Marrs, 2016
	Missed cases	Zika	Pregnant women may receive only one early pregnancy ultrasound, potentially missing the effects of infection	Brazil	Ambrogi, 2021
			80% of cases are asymptomatic	Colombia	Tirado, 2020
				Brazil; Other	Marrs, 2016
			In affected regions, many infants may have been exposed to Zika in utero without documentation of maternal exposure	Angola; American Samoa; Brazil; Columbia; Cuba; Dominican Republic; Ecuador; Guatemala; Guinea-Bissau; Haiti; Honduras; India; Jamaica; Nicaragua; Panama; Peru; Puerto Rico; Suriname; Thailand; Venezuela; Vietnam	Musso, 2019
		Ebola	Subclinical or minimally symptomatic infections in some pregnant women were not detected, hindering the identification of survivors and their long-term outcomes	Guinea	Baggi, 2014
				Sierra Leone	Lyman, 2018
