# Supplementary material for: Beyond COVID-19, the case for collecting, analysing and using sex-disaggregated data and gendered data to inform outbreak response: a scoping review
Source: BMJ Glob Health. 2025 Jan 15;10(1):e015900. doi: 10.1136/bmjgh-2024-015900 (PMC11749539; doi:10.1136/bmjgh-2024-015900)
Supplement: online supplemental file 6 [file bmjgh-10-1-s006.pdf]

Supplemental Table D. Evidence of sex, gender, and pregnancy status-related implications for treatment/management

Data Category	Theme	Disease	Evidence	Country	First Author, Year
**Treatment/management risk factors & vulnerability**					
**Sex**	No evidence identified in the literature				
**Gender**	No evidence identified in the literature				
**Pregnancy**	No evidence identified in the literature				
**Access & use of treatment/management services**					
**Sex**	No evidence identified in the literature				
**Gender**	No evidence identified in the literature				
**Pregnancy**	Acceptability	Ebola	Most recovered patients accepted that ending their pregnancy was a necessary step towards recovery	Sierra Leone	Erland, 2017
	Accessibility	Ebola	Women were unable to access healthcare services due to quarantine measures during the outbreak	Brazil; Sierra Leone; Uganda; Other	Smith, 2019
		Zika	Lack of access to medical care	Colombia; Cuba; Dominican Republic; El Salvador; Guyana; Haiti; Honduras; Mexico; Nicaragua; Other	Vlassoff, 2018
	Availability	Ebola	Fewer life-saving interventions available in Ebola treatment centres	Sierra Leone	Lyman, 2018
**Treatment/management-seeking behaviours**					
**Sex**	No evidence identified in the literature				
**Gender**	Knowledge and awareness	Ebola	Women lacked knowledge about seeking medical care during quarantine and sought care only after the 21-day period	Sierra Leone	Bower, 2016
		Influenza	Greater treatment knowledge among women (exposure to TV)	India	Gupta, 2015
	Medical pluralism	Influenza	Greater proportion of women indicated use of herbal medicine	India	Gupta, 2015
	Safety	Ebola	Treatment-seeking among women may be reduced in areas of armed conflict and insecurity due to risk of GBV	Democratic Republic of Congo	Pham, 2022
	Time to treatment	Ebola	Time from initial symptoms to hospitalisation was shorter among women	Guinea; Liberia; Sierra Leone; South Sudan; Uganda	Gomes, 2017
**Pregnancy**	Symptoms	Dengue	Roughly 76% of pregnant women sought treatment during the critical phase of infection	Indonesia	Mulyana, 2020
		Ebola	Pregnant women did not seek medical care or disclose their potential positive status due to associating symptoms with pregnancy and anxiety	Sierra Leone	Bower, 2016
**Treatment/management options**					
**Sex**	No evidence identified in the literature				
**Gender**	No evidence identified in the literature				
**Pregnancy**	Clinical guidance	Dengue	No consensus on management of infection during pregnancy	Indonesia	Mulyana, 2020
		Ebola	Lack of clinical guidance on how to care for infected pregnant women	Sierra Leone	Erland, 2017
				Brazil; Sierra Leone; Uganda; Other	Smith, 2019
			Pregnant women were excluded from drug trials	Guinea; Liberia; Sierra Leone; South Sudan; Uganda	Gomes, 2017

	Prioritisation	Dengue	Ministry of Health: pregnant women should be given priority in receiving treatment	Brazil	Nascimento, 2017
	Restricted care provision	Ebola	Infected pregnant women may not survive surgical delivery thus the use of obstetric interventions depends on mother's stability, available resuscitation efforts and the obstetric intervention in question	Guinea; Liberia; Sierra Leone; Other	Bebell, 2017
			Obstetric interventions were delayed until maternal recovery confirmed by a negative blood sample	Sierra Leone	Erland, 2017
				Sierra Leone	Oduyebo, 2015
			Due to the need for specialised care and the anticipation of poor outcomes, pregnant women were often not admitted to treatment centres	Sierra Leone	Lyman, 2018
			Foetal monitoring was not performed in treatment centres due to presumed poor foetal prognosis and concerns about healthcare worker exposure	Sierra Leone	Oduyebo, 2015
	Safety of treatment	Ebola	Convalescent plasma reduced mortality in pregnant women and a neonate born to an Ebola-positive mother survived after receiving ZMapp and GS-5734	Sierra Leone	Lyman, 2018
		Influenza	Early oseltamivir treatment linked to reduced severe disease risk; No pregnancy-related drug issues	Argentina; Brazil; South Africa; Turkey; Other	Meijer, 2015
		Malaria	WHO recommends safe antimalarial drugs for pregnant women; Drug selection should be based on regional Plasmodium species, sensitivities or resistance, potential benefits vs. risks, and contraindications during pregnancy	Burkina Faso; Gabon; Ghana; India; Kenya; Malawi; Mozambique; Nigeria; Rwanda; Senegal; Solomon Islands; Sudan; Uganda	Uneke, 2012
**Experiences in healthcare**					
**Sex**	No evidence identified in the literature				
**Gender**	Discrimination	Dengue	Women are less likely to be taken care of at hospitals when ill or are taken at late stages of disease when no other options are available	Pakistan	Aamir, 2014
		Zika	Lack of adequate medical care among women	Brazil	Ambrogi, 2021
	Gendered power dynamics	Ebola	Midwives had diverse experiences in outbreak care due to ICM restrictions; A midwife was fired for risking infection trying to improve care, but was later consulted by policymakers	Sierra Leone	Erland, 2017
**Pregnancy**	Delayed treatment	Ebola	Pregnant women were denied maternal/obstetric care until a negative blood test which resulted in vesicovaginal fistula, intrauterine foetal death, and maternal death	Sierra Leone	Erland, 2017
				Brazil; Sierra Leone; Uganda; Other	Smith, 2019
	Stigma	Zika	Stigma/judgement from doctors	Colombia	Tirado, 2020
**Health & social outcomes related to treatment/management**					
**Sex**	No evidence identified in the literature				
**Gender**	Stigma	Ebola	Community's lack of knowledge about Ebola led to fear and stigma, affecting midwives (a midwife was evicted after the landlord found out she worked in an Ebola treatment centre)	Sierra Leone	Erland, 2017
**Pregnancy**	Morbidity and mortality	Ebola	Midwives became infected and died	Sierra Leone	Erland, 2017
			Increased morbidity and mortality among women due to avoidance of health facilities	Nigeria	Fawole, 2016
