# Supplementary material for: Beyond COVID-19, the case for collecting, analysing and using sex-disaggregated data and gendered data to inform outbreak response: a scoping review
Source: BMJ Glob Health. 2025 Jan 15;10(1):e015900. doi: 10.1136/bmjgh-2024-015900 (PMC11749539; doi:10.1136/bmjgh-2024-015900)
Supplement: online supplemental file 7 [file bmjgh-10-1-s007.pdf]

Supplemental Table E. Evidence of sex, gender, and pregnancy status-related implications for recovery

Data category	Theme	Disease	Evidence	Country	First author, year
**Recovery Risk & vulnerability**					
**Sex**	Post-recovery sexual transmission	Ebola	Sexual transmission through semen post-recovery	Uganda	Kankya, 2019
				Afghanistan; Democratic Republic of Congo; Guinea; India; Liberia; Sierra Leone; Sudan; Uganda	Thorson, 2016
		Zika	RNA in semen post-recovery (prolonged potential for sexual transmission)	Colombia; Cuba; Dominican Republic; El Salvador; Guyana; Haiti; Honduras; Mexico; Nicaragua; Other	Vlassoff, 2018
				Brazil; Other	Arias, 2020
				Ecuador	Casapulla, 2018
				Brazil	Coelho, 2016
				Brazil	Calvet, 2016
			RNA has been detected in semen up to 370 days after onset of illness, but shedding of infective viral particles is rare after 30 days from the onset	Angola; American Samoa; Brazil; Columbia; Cuba; Dominican Republic; Ecuador; Guatemala; Guinea-Bissau; Haiti; Honduras; India; Jamaica; Nicaragua; Panama; Peru; Puerto Rico; Suriname; Thailand; Venezuela; Vietnam	Musso, 2019
**Gender**	No evidence identified in the literature				
**Pregnancy**	Infant fatality	Ebola	Increased risk of infant death in cases of maternal death or inability of an Ebola survivor to breastfeed	Guinea; Liberia; Sierra Leone; South Sudan; Uganda	Gomes, 2017
	Post recovery pregnancy/fertility concerns	Ebola	Increased risk for adverse foetal outcomes among cured mothers	Guinea	Baggi, 2014
				Sierra Leone	Oduyebo, 2015
				Guinea; Liberia; Sierra Leone; South Sudan; Uganda	Gomes, 2017
			Vertical transmission after cured	Guinea	Baggi, 2014
				Guinea; Liberia; Sierra Leone; South Sudan; Uganda	Gomes, 2017
			Increased risk for horizontal transmission during parturition post-maternal recovery	Guinea	Baggi, 2014
				Sierra Leone	Oduyebo, 2015
**Access & use of recovery services**					
**Sex**	No evidence identified in the literature				
**Gender**	No evidence identified in the literature				
**Pregnancy**	Availability	Ebola	Disruption to routine healthcare services, often particularly affecting maternal, child, and SRH services	Sierra Leone	Erland, 2017
				Sierra Leone	Lyman, 2018

				Brazil; Democratic Republic of Congo	Wenham, 2021
	Accessibility	Zika	Mother's often bear the burden of caring for children with CZS but face logistical barriers to obtaining medical services and welfare benefits for families with children with CZS (e.g., transportation, documentation)	Brazil	Ambrogi, 2021
				Colombia; Cuba; Dominican Republic; El Salvador; Guyana; Haiti; Honduras; Mexico; Nicaragua; Other	Vlassoff, 2018
**Health-seeking behaviours during recovery**					
**Sex**	No evidence identified in the literature				
**Gender**	No evidence identified in the literature				
**Pregnancy**	Reduced maternal health seeking behaviour	Ebola	Reduced maternal healthcare seeking behaviour due to stigma and fear of nosocomial transmission	Nigeria	Fawole, 2016
				Sierra Leone	Erland, 2017
				Sierra Leone	Lyman, 2018
**Recovery options**					
**Sex**	No evidence identified in the literature				
**Gender**	No evidence identified in the literature				
**Pregnancy**	Inadequate recovery services	Zika	Lack of medical knowledge, medications and specialised care for care of infants/children with CZS/microcephaly	Colombia	Tirado, 2020
				Brazil	Ambrogi, 2021
**Experiences with recovery services**					
**Sex**	No evidence identified in the literature				
**Gender**	No evidence identified in the literature				
**Pregnancy**	No evidence identified in the literature				
**Health & social outcomes**					
**Sex**	No evidence identified in the literature				
**Gender**	Stigmatisation	Ebola	Stigmatisation of male survivors due to fears of sexual transmission	Afghanistan; Democratic Republic of Congo; Guinea; India; Liberia; Sierra Leone; Sudan; Uganda	Thorson, 2016
	Burden of care	Ebola	Women disproportionately impacted by the burden of care after outbreaks (e.g., primary caregivers for orphans of the outbreak)	Brazil; Sierra Leone; Uganda; Other	Smith, 2019
		Zika	Women disproportionately impacted by the burden of care after outbreaks (e.g., primary and frequently sole caregivers for children with CZS)	Brazil	Ambrogi, 2021
				Colombia	Tirado, 2020
				Colombia; Cuba; Dominican Republic; El Salvador; Guyana; Haiti; Honduras; Mexico; Nicaragua; Other	Vlassoff, 2018
	Long-term economic impact	Ebola	Women disproportionately impacted by Ebola outbreak-related economic losses as they are more likely to be in less secure employment or to work in sectors of the economy shuttered by quarantine interventions	Brazil; Democratic Republic of Congo	Wenham, 2021
	Long-term mental health impact	Ebola; Zika	Women suffered increased psychological trauma, anxiety	Brazil; Sierra Leone; Uganda; Other	Smith, 2019

		Zika	and fear related to their roles and responsibilities as caregivers	Brazil; Other	Arias, 2020
	Long-term social impact	Ebola	Women who survived infection faced heightened stigma related to suspicions of witchcraft	Brazil; Sierra Leone; Uganda; Other	Smith, 2019
**Pregnancy**	Burden of care	Zika	Mother's disproportionately bear the burden or primary or sole caregiver for a child born with disabilities	Dominican Republic	Gurman, 2020
				Brazil; El Salvador; Other	Johnson, 2017
				Brazil	Sousa, 2018
				Brazil; Democratic Republic of Congo	Wenham, 2021
	Long-term economic impact	Zika	Many mothers cannot return to work after childbirth (e.g., caregiving roles, complex medical needs of their children, reliance on social welfare payments that have income restrictions)	Brazil	Ambrogi, 2021
				Brazil; Democratic Republic of Congo	Wenham, 2021
			Younger mothers faced greater barriers to accessing welfare assistance	Brazil	Ambrogi, 2021
			Children with Zika infection-related disabilities are disproportionately born to women of low SES, who may give up their jobs or studies and become single parents	Brazil; Sierra Leone; Uganda; Other	Smith, 2019
			Potential drop in birth rate due to outbreak could have national economic consequences	Brazil	Coelho, 2016
	Long-term impact on education	Zika	Closure of schools linked to increased adolescent pregnancy rates; increased gender gap in education	Brazil	Ambrogi, 2021
		Ebola; Zika		Brazil; Colombia; Ecuador; El Salvador; Guinea; Liberia; Sierra Leone	Davies, 2016
	Long-term social impact	Zika	Social isolation and stigmatisation among mothers of children with CZS	Brazil	Ambrogi, 2021
				Colombia	Tirado, 2020
			Intimate relationships with partners suffered emotionally and sexually	Brazil; Other	Arias, 2020
				Brazil; Other	Linde-Arias, 2020
			Stigma; negative and offensive comments/questions regarding infant/child with CZS	Brazil	Ambrogi, 2021
	Indirect health outcomes	Ebola	Estimated that an additional 4,022 women will die annually in childbirth in Guinea, Liberia, and Sierra Leone as a result of the Ebola-related deaths of healthcare workers in these countries	Brazil; Colombia; Ecuador; El Salvador; Guinea; Liberia; Sierra Leone	Davies, 2016
			Positive and negative women (suspected) had poor outcomes	Sierra Leone	Lyman, 2018
